# *Endozoicomonas acroporae* enhances coral thermal resilience through host–microbe coordination

**DOI:** 10.1093/ismejo/wrag173

**Published:** 2026-07-01

**Authors:** Chih-Ying Lu, Yung-Pei Chang, Victor M Piñon-Gonzalez, Thomas D Lewin, Kai-Ning Shih, Po-Shun Chuang, Naohisa Wada, Sheng-Ping Yu, Zhao-Rong Yu, Yi-Hua Chen, Mo Chen, Ching-Hsiang Chin, Yu-Jing Chiou, Yi-Ling Chiu, Isabel Jiah-Yih Liao, Hsin-Feng Chang, Jui-Hung Yen, Mei-Yeh Jade Lu, Yi-Jyun Luo, Sen-Lin Tang

**Affiliations:** Biodiversity Research Center, Academia Sinica, Taipei 11529, Taiwan; Molecular and Biological Agricultural Sciences Program, Taiwan International Graduate Program, Academia Sinica and National Chung Hsing University, Taipei 11529, Taiwan; Graduate Institute of Biotechnology, National Chung Hsing University, Taichung 40227, Taiwan; Biodiversity Research Center, Academia Sinica, Taipei 11529, Taiwan; Biodiversity Research Center, Academia Sinica, Taipei 11529, Taiwan; Biodiversity Research Center, Academia Sinica, Taipei 11529, Taiwan; Biodiversity Research Center, Academia Sinica, Taipei 11529, Taiwan; Molecular and Biological Agricultural Sciences Program, Taiwan International Graduate Program, Academia Sinica and National Chung Hsing University, Taipei 11529, Taiwan; Graduate Institute of Biotechnology, National Chung Hsing University, Taichung 40227, Taiwan; Biodiversity Research Center, Academia Sinica, Taipei 11529, Taiwan; Marine Structural Biology Unit, Okinawa Institute of Science and Technology Graduate University, Onna, Okinawa 904-0495, Japan; Biodiversity Research Center, Academia Sinica, Taipei 11529, Taiwan; Biodiversity Research Center, Academia Sinica, Taipei 11529, Taiwan; Biodiversity Research Center, Academia Sinica, Taipei 11529, Taiwan; Biodiversity Research Center, Academia Sinica, Taipei 11529, Taiwan; Biodiversity Research Center, Academia Sinica, Taipei 11529, Taiwan; Centre for Marine Science and Innovation, School of Biological, Earth, and Environmental Sciences, Faculty of Science, The University of New South Wales, Kensington, NSW 2033, Australia; Biodiversity Research Center, Academia Sinica, Taipei 11529, Taiwan; Biodiversity Research Center, Academia Sinica, Taipei 11529, Taiwan; Biodiversity Research Center, Academia Sinica, Taipei 11529, Taiwan; Biodiversity Research Center, Academia Sinica, Taipei 11529, Taiwan; Biodiversity Research Center, Academia Sinica, Taipei 11529, Taiwan; Biodiversity Research Center, Academia Sinica, Taipei 11529, Taiwan; Biodiversity Research Center, Academia Sinica, Taipei 11529, Taiwan; Molecular and Biological Agricultural Sciences Program, Taiwan International Graduate Program, Academia Sinica and National Chung Hsing University, Taipei 11529, Taiwan; Biotechnology Center, National Chung Hsing University, Taichung 40227, Taiwan; Taiwan Ocean Genome Center, National Taiwan Ocean University, Keelung 202301, Taiwan

**Keywords:** coral probiotics, coral bleaching, thermal tolerance, *Endozoicomonas*, *Stylophora pistillata*, single-cell transcriptomics

## Abstract

Probiotics hold promise for enhancing coral resilience under climate-driven thermal stress, yet their mechanisms remain poorly understood. Although the bacterial genus *Endozoicomonas* has been proposed to benefit corals, *in vivo* evidence of beneficial effects on the host remains limited. Here, we establish *Endozoicomonas acroporae* Acr-14^T^ as a coral probiotic and characterize its effects on the reef-building coral *Stylophora pistillata*. We show that *E. acroporae* Acr-14^T^ enhances host thermal tolerance, colonizes coral tissues, and forms coral-associated microbial aggregates. Microbial profiling indicates that probiotic treatment is associated with reduced relative abundances of opportunistic microbes and enrichment of putatively beneficial taxa. To support transcriptomic analyses, we assembled a chromosome-level genome of *S. pistillata* clade 1 (Pacific lineage) and found that *E. acroporae* Acr-14^T^ treatment mitigates heat-induced protein-folding stress and apoptotic signaling. Single-cell transcriptomics further revealed altered expression of genes involved in *S*-adenosylmethionine (SAMe) metabolism and pro-survival signaling in gastrodermal cells of probiotic-treated corals. Together, our results provide a cell-type-resolved view of host responses linked to *Endozoicomonas*-mediated coral thermal resilience and offer insight into molecular mechanisms implicated in host–microbe interactions under environmental stress.

## Introduction

Coral reefs are among the most biodiverse marine ecosystems on Earth, supporting over one million marine species [1] and providing ecosystem services essential to coastal economies [2]. However, more than half of the global coral cover has been lost due to anthropogenic stressors [3]. Projections indicate that if warming exceeds 1.5°C above pre-industrial levels—a threshold that may already have been surpassed [4]—up to 90% of tropical coral reefs could be lost by the end of this century [5]. The recent fourth global coral bleaching event underscores the urgency of identifying interventions that can enhance coral resilience under thermal stress [6].

Coral probiotics represent a promising strategy to improve coral health [7]. The coral probiotic hypothesis proposes that introducing beneficial microbes into reef systems can bolster coral resilience, potentially through microbiome restructuring, nutrient cycling, and the production of antioxidant or antimicrobial compounds [8]. These beneficial microorganisms for corals (BMC) [9] have been shown to mitigate bleaching [10–12] and promote recovery from heat stress, including through pathways such as dimethylsulfoniopropionate (DMSP) degradation [13]. Accumulating evidence suggests that probiotic effects are multifactorial, arising from direct host–microbe interactions, indirect microbial functions, and nutritional supplementation [14–16]. Despite these advances, the mechanistic basis of probiotic-mediated coral resilience remains incompletely understood. Progress has been limited by the complexity of the coral holobiont, the scarcity of well-annotated coral genomes, and the underutilization of high-resolution approaches such as single-cell transcriptomics [17].

*Endozoicomonas* is a widespread and abundant bacterial genus associated with healthy corals, with genomic evidence indicating that multiple species possess beneficial metabolic capacities and potential mutualistic roles [18]. However, the lack of direct experimental validation has raised uncertainty, particularly because this highly diverse genus also includes members with parasitic or pathogenic potential [19]. Previously, we reported that an *Endozoicomonas* species, *Endozoicomonas acroporae* Acr-14^T^, exhibits potent extracellular reactive oxygen species (ROS)-scavenging activity [20] and metabolizes DMSP via the cleavage pathway, thereby influencing coral sulfur cycling [21]. In addition, it carries eukaryotic-like genes containing inhibitor of apoptosis domains, which may contribute to host stress resistance [22]. However, despite these functional and genomic features, the probiotic potential of this *Endozoicomonas* strain has not been experimentally demonstrated.

Here, we evaluated the probiotic efficacy of *E. acroporae* Acr-14^T^ in *Stylophora pistillata*, a prevalent reef-building coral in which this bacterium has been detected [23, 24]. *S. pistillata* nubbins were subjected to thermal stress and inoculated with either *E. acroporae* Acr-14^T^ or filtered artificial seawater (placebo). We monitored coral bleaching responses, microbial community dynamics, and host transcriptomes at both bulk and single-cell resolution. Our objectives were to evaluate the probiotic potential of *E. acroporae* Acr-14^T^ and to characterize microbial and transcriptional signatures associated with enhanced thermal resilience.

## Materials and methods

### Fieldwork

All corals used in this study belong to *S. pistillata* clade 1*.* Nubbins were collected from Kenting, Taiwan (21.930240°N, 120.745618°E; Fig. S1A) under permits no. 1110006783. For *S. pistillata* clade 1 genome sequencing, a single colony was collected from the same site. Detailed sampling methods and environmental conditions are provided in the Supplementary Materials.

### Treatment setup and sampling strategies

**Figure 1:**
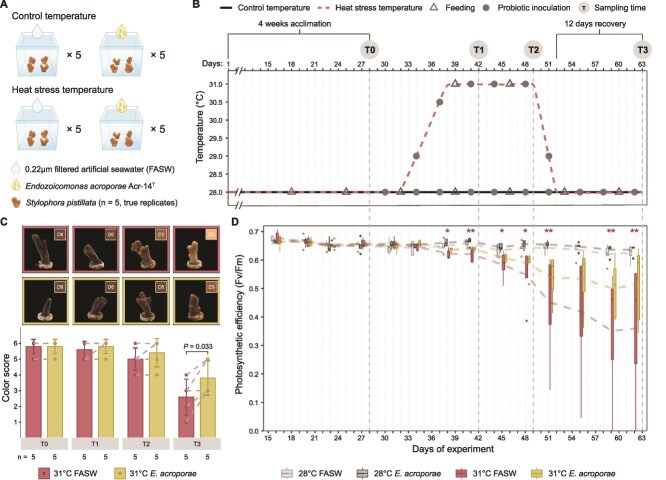
*Endozoicomonas acroporae* attenuates coral bleaching responses. (A) Treatment and replication design for the coral bleaching experiment. Each treatment comprised five independent aquarium systems, with each aquarium containing four coral nubbins assigned to different sampling time points. At each sampling time point, five nubbins per treatment were collected, each originating from a different coral colony (*n* = 5). (B) Treatment regimens, including temperature settings and the timing of feeding, probiotic inoculation, and sample collection. (C) Coral color scores of nubbins collected at four sampling time points, with representative photographs of nubbins from colony c. Gray dashed lines connect data from the same colony across treatments. Dots represent individual data points. Data are shown as means ± SD (*n* = 5; two-sided paired *t-*test). (D) Photosynthetic efficiency (Fv/Fm) of coral nubbins. In the boxplots, dots represent outliers, and dashed lines connect treatment means (*n* = 5; one-way ANOVA with Tukey’s post hoc test; ^*^31°C FASW significantly lower than 28°C *E. acroporae*; ^**^31°C FASW significantly lower than both 28°C FASW and 28°C *E. acroporae*; adjusted *P* < .05; Table S1C).

Upon arrival at the laboratory, 80 coral nubbins from five colonies were distributed across 20 aquaria. Four treatments were established, each comprising five independent aquaria, with aquaria serving as the unit of replication (*n* = 5 per treatment; Fig. S1C). Treatments were (i) ambient temperature with FASW (28°C FASW), (ii) ambient temperature with *E. acroporae* Acr-14^T^ (28°C *E. acroporae*), (iii) heat stress with FASW (31°C FASW), and (iv) heat stress with *E. acroporae* Acr-14^T^ (31°C *E. acroporae*; Fig. 1A). Samples were collected at four time points: day 28 (post-acclimation, T0), day 42 (early heat stress, T1), day 49 (peak heat stress, T2), and day 63 (post-recovery, T3; Fig. 1B). Detailed aquarium setup, maintenance conditions, and sampling procedures are provided in the Supplementary Materials.

### Probiotic treatment preparation and inoculation

*Endozoicomonas acroporae* Acr-14^T^ was originally isolated from *Acropora* sp. in Kenting, Taiwan [25]. Bacteria were cultured in Modified Marine Broth Version 4 (MMBV4) medium [26]. Cultures were harvested by centrifugation and resuspended in FASW. Probiotics were inoculated into coral tanks twice weekly to a final OD_600_ of 0.1, corresponding to 1.19 ± 0.64 × 10^7^ viable cells/ml (Fig. S2). Control tanks received an equivalent volume of FASW as placebo. Detailed inoculation procedures are provided in the Supplementary Materials.

### Physiological analyses

Coral bleaching responses were monitored using pulse-amplitude-modulated (PAM) fluorometry (junior-PAM system, Walz GmbH). Nubbin coloration was assessed using the Coral Health Chart [27]. Antioxidant enzyme activities, including catalase (CAT) and superoxide dismutase (SOD), were measured spectrophotometrically. Detailed PAM parameter settings and enzyme activity measurement procedures are provided in the Supplementary Materials.

### Microbiome analysis

Bacterial communities were assessed using 16S rRNA gene amplicon sequencing (V6–V8) and PacBio full-length 16S rRNA gene amplicon sequencing. Data were processed using QIIME2 and DADA2 to obtain amplicon sequence variants. Decontamination was performed using blank controls. To visualize probiotic colonization, FISH was performed on paraffin-embedded sections using an *Endozoicomonas*-specific probe with QuickHCR [28] signal amplification. Detailed DNA extraction method, primer sequences, PCR conditions, bioinformatics pipelines, and FISH protocols are provided in the Supplementary Materials.

### Genome assembly and bulk RNA-seq

To facilitate transcriptomic analysis, a chromosome-level *S. pistillata* clade 1 genome was generated using PacBio HiFi sequencing and Hi-C scaffolding. For bulk RNA-seq, libraries were sequenced on the NextSeq 2000 sequencing system (Illumina). Trimmed reads were aligned to the reference genomes (*S. pistillata* clade 1 for the coral host and *Cladocopium goreaui* for the symbiotic algae) using HISAT2. Transcript abundance was estimated using StringTie. Differential gene expression analysis was performed using DESeq2. Gene Ontology (GO) enrichment and gene set enrichment analysis (GSEA) were conducted using the clusterProfiler package. Detailed sample preparation procedures, DNA/RNA extraction methods, and bioinformatic pipelines are provided in the Supplementary Materials.

### Single-cell RNA-seq

To facilitate single-cell transcriptomic analysis, we employed a modified fixation-compatible workflow. Dissociated cells were fixed using an acetic acid–methanol (ACME) protocol optimized for the 10x Genomics platform. Data were processed using Cell Ranger. Background RNA contamination was mitigated using CellBender. Noise-removed matrices were imported into Seurat. Doublets were identified and removed using the scDblFinder package. Clusters were defined using the FindNeighbors and FindClusters functions with a resolution of 0.6. Cell types were annotated via orthology-based comparison with the existing *S. pistillata* clade 4 cell atlas [29]. Detailed dissociation procedures, fixation protocol, and bioinformatic pipelines are provided in the Supplementary Materials.

### Statistical analysis

Specific statistical tests used for each analysis are indicated in the Supplementary Materials or the corresponding figure legends. Sample sizes or gene numbers for each analysis are indicated by “*n*” in corresponding figure panels or legends.

## Results

### *Endozoicomonas acroporae* Acr-14^T^ confers thermal resilience on *S. pistillata*

Throughout the experiment, ambient-temperature nubbins maintained stable photosynthetic efficiency regardless of treatment (Fig. 1D). In the 31°C *E. acroporae* treatment, photosynthetic efficiency declined slightly but remained comparable to that of the ambient controls (adjusted *P* > .05). In contrast, the 31°C FASW group showed significantly lower photosynthetic efficiency than both ambient controls beginning during early heat stress, with significant differences detected from day 41 and at most subsequent time points (adjusted *P* < .05). This decline coincided with visible bleaching and reduced color scores compared to the 31°C *E. acroporae* treatment at T3 (*P* < .05; Fig. 1C).

### *Endozoicomonas acroporae* physically colonizes host tissue and forms microbial aggregates

**Figure 2:**
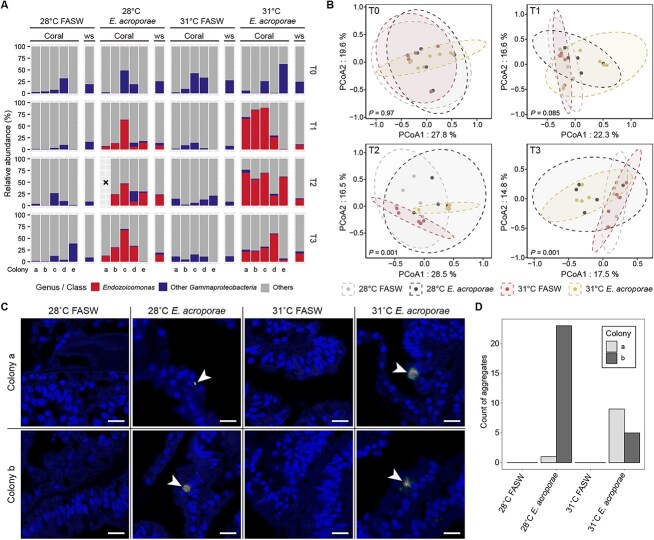
*Endozoicomonas acroporae* colonizes host tissues and forms CAMAs. (A) Relative abundance of *Endozoicomonas* and other *Gammaproteobacteria* in samples via short-read 16S rRNA gene amplicon sequencing. Each bar represents one sample, with coral colony codes indicated on the *x*-axis. Tank water samples, ws; lost sample, x. (B) Principal coordinates analysis (PCoA) illustrating treatment effects on coral microbial community composition at each time point. Ellipses represent 95% confidence intervals around group centroids (PERMANOVA; pairwise comparison *P*-values adjusted using Bonferroni correction). (C) Representative confocal images showing merged fluorescence in situ hybridization (FISH) signals using the EUB338mix probe [68] (red, all bacteria), the Endo-Group B probe [39] (green, *Endozoicomonas-*specific), and the Non338 probe [69] (white, negative control, no signal detected). DAPI staining (blue) marks nuclei and host autofluorescence. White arrows indicate localized *Endozoicomonas* signals in coral tissues at T3. Scale bars, 10 μm. (D) Quantification of *Endozoicomonas* aggregates across the four treatments at T3 (30 tissue sections were observed per treatment).

We first examined whether *E. acroporae* Acr-14^T^ actively colonizes coral hosts and forms a stable microbial association. Microbiome profiling based on 16S rRNA gene amplicon sequencing showed that coral microbial communities were dominated by *Alphaproteobacteria, Gammaproteobacteria, Desulfovibrionia*, and *Campylobacteria* (Fig. S3A). *Endozoicomonas acroporae* was consistently detected in corals receiving the probiotic treatment (Figs 2A and S3B), whereas probiotic-untreated corals, including baseline samples (T0), contained either no or very low (< 0.5%) *Endozoicomonas* sequences (Fig. 2A).

To determine whether *E. acroporae* Acr-14^T^ physically associates with the coral host, we performed fluorescence in situ hybridization (FISH) on T3 samples. *Endozoicomonas*-specific signals were detected exclusively in treated corals (Fig. 2C and D). These bacteria formed coral-associated microbial aggregates (CAMAs) in the tissue, the number and size of which correlated with the relative abundance of *E. acroporae* Acr-14^T^ (Fig. S4B and C). Signals were predominantly localized to mesenterial filaments (Figs S4A and S5), with fewer detected in tentacles or coenosarc, similar to observations in wild corals [30]. Collectively, these results demonstrate that probiotic treatment establishes a stable, tissue-associated host–microbe interaction.

### Probiotic treatment is associated with shifts in opportunistic and beneficial microbes

We analyzed microbial composition to determine how probiotic treatment and heat stress affected the coral microbiome. Probiotic treatment had a limited impact on microbial alpha diversity (Fig. S3C), whereas microbial composition exhibited clear temporal dynamics (Fig. 2B). No significant differences were observed among treatments at baseline (T0) or during early treatment initiation (T1). At peak heat stress (T2), significant compositional differences emerged between the 31°C FASW and 31°C *E. acroporae* treatments (adjusted *P* = .03). After recovery (T3), differences were primarily detected between the 28°C FASW and 31°C *E. acroporae* groups (adjusted *P* = .048). These patterns suggest that specific community structures reflect responses to probiotic and heat stress treatments.

**Figure 3:**
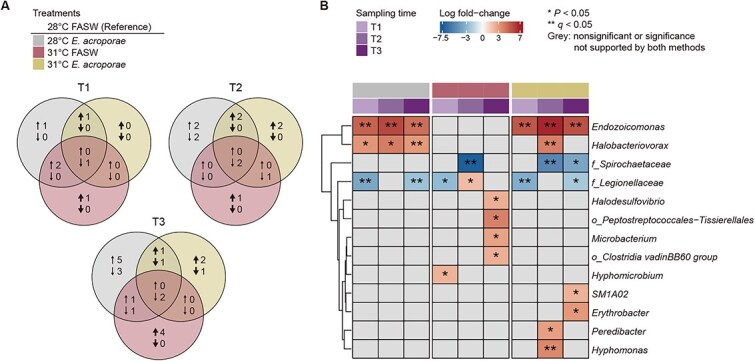
Differential abundance of bacterial genera in coral samples among treatments. (A, B) Only genera identified as significantly different by both methods (ANCOM-BC and MaAsLin2; *P* < .05) are shown. Venn diagrams summarize differential abundance results across treatments, indicating enriched and depleted genera, with genera of particular interest highlighted by thick arrows (A). Heatmaps illustrate effect sizes for selected genera (B). Gray grids indicate *P* > .05 or results not supported by both methods. ^*^P < .05; ^**^q < .05, adjusted by Holm–Bonferroni correction. Detailed statistics are provided in Table S3.

To identify microbial taxa associated with *E. acroporae* Acr-14^T^ treatment, we performed genus-level differential abundance analyses using a consensus approach combining ANCOM-BC and MaAsLin2 (Supplementary Materials). Using the 28°C FASW control as a reference, we focused on genera uniquely associated with either the 31°C FASW or 31°C *E. acroporae* treatments (Fig. 3A). Opportunistic taxa including *Legionellaceae* (genus unassigned) [31] were enriched at T2, while *Halodesulfovibrio* [32] and *Peptostreptococcales-Tissierellales* (genus unassigned) [33] were enriched at T3 in the 31°C FASW treatment (Figs 3B and S6). In contrast, the putatively beneficial microbe *Halobacteriovorax* [34] was significantly enriched in *E. acroporae*–treated corals (Figs 3B and S6). This association was further supported by co-occurrence network analysis, which revealed a positive correlation between *Endozoicomonas* and *Halobacteriovorax* (Fig. S7), suggesting coordinated microbial interactions. Together, these results indicate that *E. acroporae* Acr-14^T^ is associated with reduced abundance of opportunistic taxa and increased persistence of beneficial microbes.

### *Endozoicomonas acroporae* is associated with modulation of protein-folding and apoptotic pathways

To enable transcriptomic analysis of probiotic effects, we generated a chromosome-level genome assembly for the *S. pistillata* clade (clade 1) used in this study, which substantially improved transcriptome alignment efficiency compared with mapping to the clade 4 (Red Sea lineage) reference (Supplementary Results; Fig. S8D). Profiling coral host and algal symbiont transcriptomes showed that, relative to ambient controls (28°C FASW), most differentially expressed genes (DEGs) in the 28°C *E. acroporae* treatment appeared at T1, whereas the strongest responses in heat-stressed corals occurred at T2 (Figs S9A and S10A). Overall host gene expression profiles were largely colony-dependent (Fig. S9B), whereas symbiont expression reflected both colony and heat-stress effects (Fig. S10B). When focusing on DEGs at each time point, host expression profiles at T2 showed clear modulation by probiotic treatment under heat stress (Fig. S9C), a pattern absent in the symbiont (Fig. S10C). These results suggest that the transcriptomic response to *E. acroporae* Acr-14^T^ is more pronounced in the coral host than in the algal symbiont.

**Figure 4:**
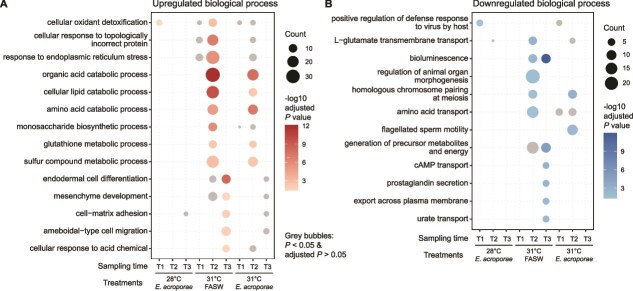
*Endozoicomonas acroporae* modulates coral host transcriptional responses to heat stress. (A, B) GO enrichment analyses of upregulated (A) and downregulated (B) DEGs across treatments and time points. The top five biological processes (sorted by adjusted *P*-value) identified in each treatment are shown. Bubble size reflects DEG count. Gray bubbles represent GO terms with *P* < .05 but adjusted *P* > .05 (Benjamini–Hochberg correction).

GO enrichment analysis of the host transcriptome revealed that heat stress (31°C FASW) upregulated genes involved in oxidant detoxification, protein folding, response to endoplasmic reticulum stress, and nutrient metabolism (Fig. 4A). By T3, DEGs shifted toward pathways associated with cell differentiation and matrix adhesion. These responses were attenuated in probiotic-treated corals, particularly in pathways related to protein folding and endoplasmic reticulum stress. Additionally, heat stress suppressed genes involved in glutamate transport, meiosis, and energy production, whereas probiotic treatment mitigated many of these suppressive effects (Fig. 4B). Direct comparison between the two 31°C treatments revealed relatively few DEGs (*n* = 114; Fig. S9D), with two immune-associated genes, *ANGPTL7* and *VWF*, significantly downregulated in the 31°C *E. acroporae* treatment. However, GSEA, which detects coordinated pathway-level changes [35], uncovered consistent downregulation of stress-related pathways in the probiotic-treated group: viral defense at T1, misfolded protein response at T2, and apoptotic signaling at T3 (Fig. S9E and F). At T1, reduced expression of genes associated with innate immune-related pathways, including several GO terms related to viral defense, was detected in probiotic-treated corals (Fig. 4B). These pathways included genes such as *CGAS, JAK3*, and *DTX3L*, many of which were identified as DEGs at T1 but not at later time points (Fig. S11). This suggests that probiotic-associated immune modulation was most pronounced at the early stage and became attenuated over time. Together, these results indicate that *E. acroporae* Acr-14^T^ treatment was associated with early immune modulation across temperature conditions, whereas under heat stress it was associated with reduced activation of host stress-related pathways, including those related to protein folding and apoptosis.

For the algal symbiont transcriptome, comparison using 28°C FASW as a reference detected no significant GO enrichment in the 28°C *E. acroporae* group (Fig. S10D and E). In heat-stressed treatments, functions enriched among upregulated genes at T2 included photosynthesis and chlorophyll biosynthesis (Fig. S10D), whereas downregulated genes were associated with nitrate assimilation and peptide biosynthesis (Fig. S10E). Significant enrichment of downregulated genes related to ammonium transport was observed only in the 31°C FASW group, consistent with increased ammonium availability in the holobiont. Few DEGs and GSEA signals were detected in the direct comparison between the two 31°C treatments (Fig. S10F and G), with genes encoding glutamate synthase and glutathione S-transferase specifically upregulated at T2 in the 31°C *E. acroporae* treatment.

To complement transcriptomic analyses, we measured catalase (CAT) and superoxide dismutase (SOD) activities in both coral hosts and their symbiotic algae. During heat stress, coral host CAT activity increased significantly in both 31°C treatments compared to the 28°C FASW control (Fig. S12A). Algal symbionts also exhibited elevated CAT activity under heat stress, although a significant increase was only observed at T1 in the 31°C *E. acroporae* group (Fig. S12A). Symbiont SOD activity showed an increasing trend under heat stress, but differences between treatments were not statistically significant at any time point (Fig. S12B). Coral host CAT activity was also significantly higher in the 28°C *E. acroporae* treatment than in the 28°C FASW control at T1 (Fig. S12A), consistent with transcriptomic evidence for upregulation of genes involved in oxidant detoxification (Fig. 4A).

### A single-cell atlas of *S. pistillata* clade 1 reveals 31 coral cell types

**Figure 5:**
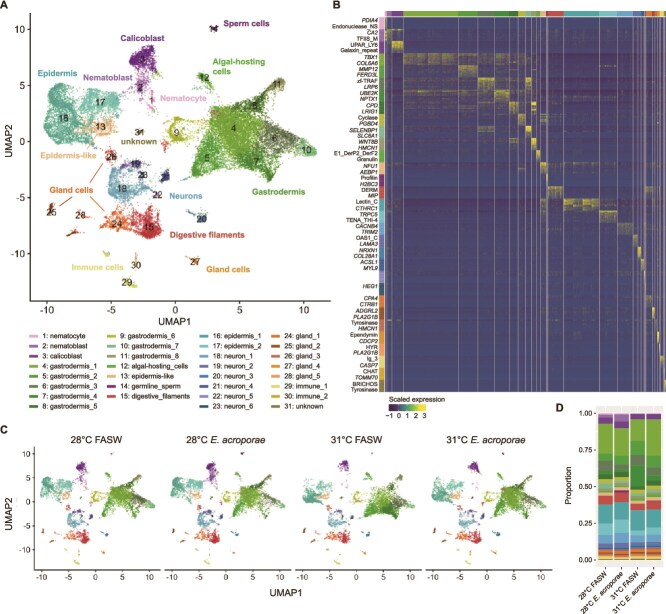
A single-cell atlas of *S. pistillata* clade 1 identifies 31 coral cell types. (A) Two-dimensional projection of *S. pistillata* clade 1 single-cell transcriptomes, showing 31 cell clusters across 29 637 cells. (B) Normalized expression of the top 10 marker genes (ranked by averaged log_2_ fold change) for each cell cluster (adjusted *P* < .05). Cell types are color-coded on both axes, and the x-axis represents individual cells. Marker genes with annotated gene names or Pfam domains are labeled on the *y*-axis (maximum of two labels per cluster; Table S4). (C) Cell-type atlas of *S. pistillata* clade 1 across all treatments at T2. (D) Proportions of each cell cluster across treatments at T2.

To characterize probiotic treatment effects at single-cell resolution, we performed single-cell RNA sequencing (scRNA-seq) on four samples, each representing three coral colonies per treatment at T2 (Supplementary Materials). After filtering ambient background RNA, doublets, and low-quality cells, we detected 35 530 genes across all samples, slightly more than the 33 346 genes detected by bulk RNA-seq from the same colonies. Dimensionality reduction using PCA and UMAP revealed 31 distinct cell clusters in *S. pistillata* clade 1, each defined by uniquely expressed marker genes (Fig. 5A and B; Table S4).

To assign cell-type identities, we used 491 previously defined marker genes from the *S. pistillata* clade 4 cell atlas [29], identifying 280 orthologous markers in clade 1 through reciprocal best hits (RBH; Table S5A). Among these, 181 highly variable markers (average log2 fold-change >1.7) were used to classify cell clusters. Most clusters were clearly resolved (Fig. S13), though cluster 13, initially identified as mitotic host cells, shared several markers with epidermal cells and was reclassified accordingly. Cluster 31 expressed markers associated with multiple cell types and remained unassigned (Fig. S13). Nematocytes specifically expressed *POU4F3*, a gene previously linked to these differentiated stinging cells [36], whereas their precursors, nematoblasts, shared several markers with nematocytes, but additionally expressed *CA2*, a gene associated with calicoblasts (Fig. S14A). Functional and evolutionary profiles of these annotated cell types further support their cell-type assignments (Supplementary Results; Fig. S14B–F). Together, this single-cell atlas provides the first high-resolution view of *S. pistillata* clade 1 cell diversity, establishing a framework to explore how specific cell types contribute to probiotic responses under thermal stress.

### Cell type-specific heat stress responses under probiotic treatment

We examined cell-type composition in *S. pistillata* across temperature and probiotic treatments (Fig. 5C and D). Cell-type distributions differed significantly among treatments (Chi-squared test, *P* < 2.2e-16). At 31°C, FASW corals showed reduced nematoblasts and an increased proportion of gastrodermis_4 (Fig. S15A). In contrast, *E. acroporae*–treated corals did not show an increase in gastrodermis_4, and instead showed a reduced proportion of nematoblast cells and an increased proportion of germline_sperm cells. The 28°C *E. acroporae* treatment also showed a significant increase in germline_sperm (Fig. S15A). These results indicate that *E. acroporae* treatment is associated with maintenance of gastrodermal cell-type proportions and altered germline cell-type distributions under heat stress.

To investigate transcriptomic responses at single-cell resolution, we performed differential expression analyses using the 28°C FASW control as a reference. The 31°C FASW treatment yielded the highest number of DEGs, whereas the 28°C *E. acroporae* treatment induced relatively few (Fig. S15B). Across all treatments, most DEGs were detected in gastrodermal and epidermal cell populations (Fig. S15B), highlighting their sensitivity to environmental and probiotic influences. To validate these single-cell signatures, we cross-referenced them with our bulk RNA-seq dataset, which provided high biological replication (*n* = 4). Pseudobulk scRNA-seq gene expression levels were highly correlated with those from bulk RNA-seq across all four treatments (Rs = 0.817–0.825; Fig. S15C), confirming that the single-cell dataset accurately captures the colony-level transcriptomic landscape.

**Figure 6:**
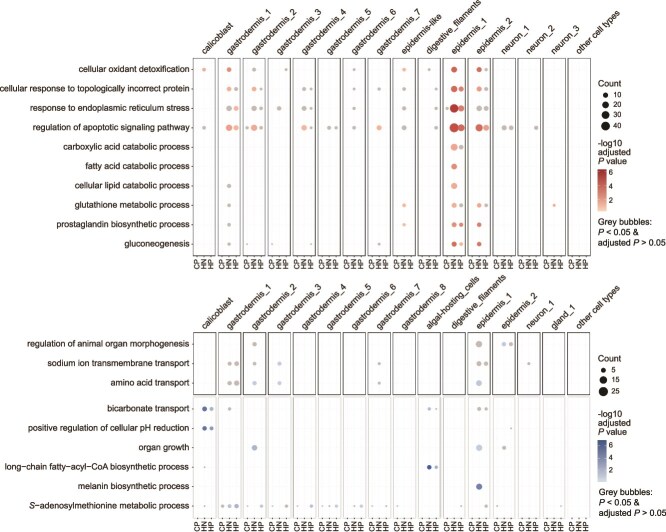
*E. acroporae* modulates heat stress-associated transcriptional responses across coral cell types. GO enrichment analysis of upregulated (upper panel) and downregulated (lower panel) DEGs in each cell cluster across treatments. Black boxes highlight significantly enriched GO terms (adjusted *P* < .05) detected in both bulk and single-cell transcriptomic dataset, whereas gray boxes indicate terms identified only in the single-cell dataset. Bubble size represents DEG count. Gray bubbles indicate GO terms with *P* < .05 but adjusted *P* > .05 (Benjamini–Hochberg correction). CP: 28°C *E. acroporae*; HN: 31°C FASW; HP: 31°C *E. acroporae*.

Leveraging this concordance, we utilized the bulk data to ensure statistical rigor and the single-cell atlas to deconvolute the cellular origin of these signatures. We restricted our single-cell GO enrichment analysis to significant GO terms that were also detected in bulk RNA-seq data or supported by previous heat stress-related studies. In the 31°C FASW treatment, GO terms associated with heat stress, such as response to unfolded proteins, endoplasmic reticulum stress, and regulation of apoptotic signaling, were significantly upregulated and enriched in multiple gastrodermal and epidermal cell types (Fig. 6). In contrast, metabolic processes, including lipid catabolism and gluconeogenesis, were specifically enriched in epidermal cells (Fig. 6). These stress-induced responses were attenuated in the 31°C *E. acroporae* treatment.

GO enrichment analysis of downregulated DEGs revealed fewer overlaps with bulk RNA-seq results but identified additional cell type-specific responses. Genes related to bicarbonate transport and intracellular pH regulation, processes critical for coral calcification, were downregulated in calicoblasts under heat stress (Fig. 6). In algal-hosting cells, DEGs associated with long-chain fatty-acyl-CoA biosynthesis were downregulated, whereas genes involved in melanin biosynthesis were downregulated in epidermis_1 under heat stress, suggesting disruption of protective pigmentation pathways (Fig. 6). In gastrodermal cells, the SAMe metabolic process was enriched among downregulated DEGs under both ambient and heat-stressed conditions in *E. acroporae*–treated corals.

### *RET* and *MAT1A* show treatment-specific regulation under *E. acroporae* exposure

**Figure 7:**
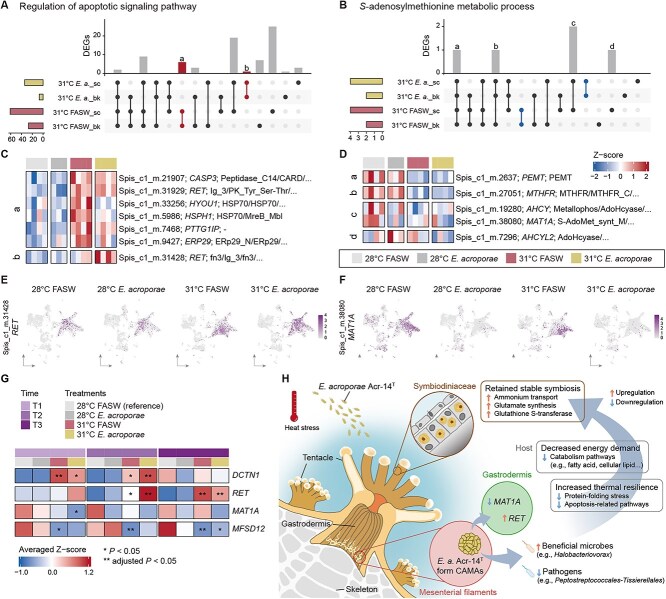
*E. acroporae*–associated thermal resilience is linked to transcriptional upregulation of *RET* and reduced *MAT1A* expression. (A, B) Overlaps of DEGs in the 31°C FASW and 31°C *E. acroporae* treatments (both relative to 28°C FASW), associated with GO terms for the regulation of apoptotic signaling pathway (A) and the *S*-adenosylmethionine metabolic process (B). DEGs identified by bulk (“bk”) or single-cell (“sc”) transcriptomics are indicated*.* Letters on the bar charts correspond to the specific genes shown in panels (C) and (D), with colors highlighting DEGs uniquely detected in either the 31°C FASW or 31°C *E. acroporae* treatment. (C and D) Bulk RNA-seq expression of selected genes associated with the regulation of apoptotic signaling pathway (C) and the *S*-adenosylmethionine metabolic process (D). Annotated gene names and Pfam domains are listed after gene IDs. (E and F) Normalized single-cell RNA-seq expression of *RET* (E) and *MAT1A* (F). (G) Heatmap showing z-scaled bulk transcriptome expression of genes potentially contributing to the probiotic effect; asterisks indicate statistical significance from DESeq2. (H) Schematic summarizing the proposed model of how *E. acroporae* Acr-14^T^ enhances the thermal resilience of *S. pistillata*.

To identify candidate genes mediating the observed probiotic effects, we examined DEGs involved in processes including protein folding, apoptotic signaling, melanin biosynthesis, and the SAMe metabolic pathway. We focused on DEGs uniquely detected in either the 31°C FASW or 31°C *E. acroporae* treatments that were consistently identified in both bulk and single-cell transcriptomes. In the 31°C FASW treatment, genes associated with the unfolded protein response, including *PARG, HSPH1*, and *ATP2A2*, were significantly upregulated (Fig. S16A and C). In contrast, *DCTN1*, which is involved in stress granule disassembly in heat-stressed cells [37], was uniquely upregulated in the 31°C *E. acroporae* treatment (Fig. S16C and E). Consistently, pro-apoptotic genes including *CASP3, HYOU1*, and *ERP29* were upregulated only under heat stress without probiotic treatment, whereas *RET*, a gene associated with cell-survival signaling, was specifically upregulated in the probiotic-treated group (Fig. 7A, C, and  E).

Genes involved in melanin biosynthesis, such as *MFSD12*, were downregulated exclusively in the 31°C FASW treatment (Fig. S16B, D, and  F). No SAMe pathway-associated DEGs were uniquely detected in either treatment group across both transcriptomes (Fig. 7B and D). However, single-cell transcriptomic analysis revealed pronounced downregulation of *MAT1A*, a key gene involved in SAMe synthesis, in both the 28°C and 31°C *E. acroporae* treatments (Fig. 7F), indicating a strong and consistent transcriptional response to probiotic exposure.

As single-cell transcriptome data represent only T2, we queried bulk transcriptome data from T1 to T3 to determine whether these gene expression patterns persisted throughout the experiment. *DCTN1* was significantly upregulated in the 31°C *E. acroporae* treatment at T2, but its expression level at T1 was lower than in the 31°C FASW treatment (Fig. 7G). In contrast, *RET* expression during heat stress remained consistently higher in the 31°C *E. acroporae* group, whereas *MAT1A* expression was persistently lower in probiotic treatments compared with their corresponding FASW controls, indicating a sustained probiotic effect (Fig. 7G). Collectively, these findings identify treatment-specific regulation of stress-associated genes, including *RET* and *MAT1A*, in *E. acroporae*–treated corals under heat stress.

## Discussion

Coral probiotics can bolster thermal resilience [10, 16], yet their molecular underpinnings remain underexplored. Here, we demonstrate that *E. acroporae* Acr-14^T^ functions as an effective coral probiotic that enhances thermal tolerance in *S. pistillata*. In the following sections, we discuss *E. acroporae* Acr-14^T^ influence on coral microbial communities, the molecular pathways that may contribute to enhanced coral thermal resilience, and its application potential.

### Establishment of CAMAs and beneficial restructuring of the coral microbiome

Our results show that *E. acroporae* Acr-14^T^ establishes symbiosis and forms CAMAs in *S. pistillata* tissues, similar to those observed in *Acropora kenti* [38]. In natural reef environments, CAMAs are commonly formed by *Endozoicomonas*, which can be highly abundant in corals. For example, its relative abundance can exceed 90% in *S. pistillata* from Kenting, Taiwan [39]. However, *Endozoicomonas* abundance was very low in the baseline samples (T0) of the present experiment after four weeks of acclimation under artificial culture conditions, consistent with previous reports of marked *Endozoicomonas* declines in corals maintained in aquarium environments [40, 41]. This low baseline abundance provided a useful context to test whether *E. acroporae* Acr-14^T^ could re-establish tissue-associated CAMAs, thereby offering a tractable model for investigating the functional roles and molecular mechanisms of *Endozoicomonas*-coral symbiosis. Genomic analyses indicate that *Endozoicomonas* genomes are enriched in eukaryotic-like proteins (ELPs) [42], with ephrin ligand genes proposed to mediate host interaction [22, 43]. However, *E. acroporae* Acr-14^T^ and related strains lack these coral-like ephrin ligand genes, suggesting alternative colonization strategies [22]. Our bulk transcriptomic data revealed significant downregulation of innate immune genes during early treatment initiation, including *CGAS*, in corals treated with *E. acroporae* Acr-14^T^. Immune modulation may therefore represent one strategy by which *Endozoicomonas* facilitates symbiosis.

Microbiome restructuring is a common feature of coral probiotic action [14]. Such shifts may arise from antimicrobial production [44], competitive exclusion [10, 13], ROS scavenging [45], or modulation of host immunity [11], thereby steering the microbiome toward a beneficial state. *Endozoicomonas* abundance often correlates negatively with coral stress or disease [46, 47], a pattern mirrored in our study, where *E. acroporae* Acr-14^T^ suppressed opportunistic pathogens associated with stony coral tissue loss disease (SCTLD), including *Halodesulfovibrio* and *Peptostreptococcales-Tissierellales* [32, 33], as well as thermal stress-associated *Legionellaceae* [31]. Functionally, these shifts may be driven in part by regulation of oxidative stress. *E. acroporae* Acr-14^T^ exhibits strong extracellular ROS-scavenging capacity [20], which may help maintain redox homeostasis and limit niches favorable to opportunistic pathogens. Direct measurements of ROS levels (e.g. DCFH-DA assays) could further clarify the extent of redox modulation mediated by *E. acroporae* Acr-14^T^. Beyond pathogen suppression, *E. acroporae* Acr-14^T^ also promoted health-associated taxa, such as *Halobacteriovorax*, a predatory bacterial genus known to control *Vibrio* infections in corals [34, 48]. Such restructuring of the microbial network may foster interactions that outcompete or prey upon pathogens, thereby enhancing coral resilience. Future co-culture experiments involving *E. acroporae* Acr-14^T^ and *Halobacteriovorax* could help elucidate their functional interplay and inform the design of optimized probiotic consortia.

### Cellular insights into coral thermal stress

We found that coral heat stress responses were primarily localized to gastrodermal and epidermal cells, consistent with prior studies showing heat stress-associated gene expression in these tissues [49]. Histological observations further support this conclusion, revealing pronounced gastrodermal tissue damage under heat stress [50]. We also observed an increased proportion of gastrodermis_4 cells in heat-stressed corals, a shift that was absent in the *E. acroporae*–treated group. This finding suggests that *E. acroporae* Acr-14^T^ treatment may attenuate heat stress impacts in the gastrodermis, consistent with our functional enrichment analyses.

Single-cell transcriptomic data further revealed responses characteristic of aposymbiotic corals. In algal-hosting cells, heat stress significantly downregulated the long-chain fatty-acyl-CoA biosynthetic pathway, which is essential for lipid synthesis and energy metabolism. Because Symbiodiniaceae supply fatty acids to the coral host [51], reduced fatty acid availability during bleaching may contribute to this suppression. We also identified a heat-induced decline in melanin expression, a key component of innate immunity in cnidarians [52]. Although decreased melanin levels during bleaching have been reported previously [53], our results localize this reduction specifically to epidermal cells. Additionally, calcification-related processes were suppressed in calicoblasts under heat stress. This observation aligns with a recent single-cell study of *Orbicella faveolata* [54], which also documented a reduced proportion of cnidocytes under heat stress. In the *S. pistillata* model, we further resolved this decline to nematoblasts, a pattern that was not rescued by *E. acroporae* Acr-14^T^ treatment, indicating high thermal sensitivity of this cell type.

### Mechanisms underlying coral thermal resilience conferred by *E. acroporae*

Heat-induced coral bleaching involves multiple factors, including elevated temperature-driven ROS accumulation, which promotes protein misfolding and endoplasmic reticulum stress and ultimately contributes to bleaching and apoptosis [55]. Coral probiotics selected for strong ROS-scavenging capacity have been shown to reduce ROS levels [9, 45] and suppress apoptotic signaling [13] in heat-stressed corals. Our results indicate that *E. acroporae* Acr-14^T^ is associated with elevated coral host catalase activity and upregulation of a symbiont ROS-detoxification gene, glutathione S-transferase, which may help alleviate oxidative and protein-folding stress in heat-stressed *S. pistillata*. In addition, *E. acroporae* Acr-14^T^ possesses the capacity to degrade DMSP via the cleavage pathway, producing dimethyl sulfide (DMS) and 3-hydroxypropionate [21]. DMS is more reactive toward ROS than DMSP itself [56], suggesting an additional route for oxidative stress mitigation. Moreover, *E. acroporae* Acr-14^T^ may contribute to host stress tolerance by carrying genes capable of suppressing apoptosis triggered by innate immune pathways [22]. This interpretation is consistent with the downregulation of innate immune and apoptotic signaling pathways detected in the 31°C *E. acroporae* treatment.

Beyond ROS-mediated effects, our results suggest that the enhanced thermal tolerance associated with *E. acroporae* Acr-14^T^ may involve coordinated host transcriptional responses, rather than antioxidant activity alone. Heat stress can also disrupt nutrient cycling between corals and their algal symbionts. Elevated host energy demand under heat stress activates catabolic pathways that shift nitrogen balance from net uptake to ammonium release, reducing carbon transfer and destabilizing the symbiosis [57]. Our bulk transcriptome analysis showed that significant downregulation of the symbiont ammonium transport pathway occurred exclusively in the 31°C FASW treatment. This pattern may reflect *E. acroporae*–associated thermal protection that helps maintain host energy homeostasis, as evidenced by the absence of enriched downregulated genes related to energy production and only minor activation of catabolic pathways in probiotic-treated corals. Such regulation may limit ammonium release and help preserve the coral–algal symbiosis under heat stress.

Single-cell transcriptomic analysis revealed significant upregulation of *RET* and downregulation of *MAT1A* in *E. acroporae*–treated corals. Given *RET*’s established role in promoting cell survival and stress tolerance [58], its early activation may help dampen coral stress responses during thermal exposure. *MAT1A* encodes an enzyme responsible for SAMe synthesis and is homologous to *sams-1* in *Caenorhabditis elegans* [59]. Previous work showed that *sams-1* knockdown enhances thermal tolerance in *C. elegans* [60], potentially through altered one-carbon metabolism [61] or phosphatidylcholine synthesis [62]. By analogy, *E. acroporae* Acr-14^T^ may confer similar benefits in corals through *MAT1A* downregulation*.* Although *sams-1* knockdown also increases pathogen susceptibility in *C. elegans* [63], this effect was not observed in corals treated with *E. acroporae* Acr-14^T^, suggesting that probiotic association may mitigate the trade-off between stress tolerance and immune defense. Future functional validation of *MAT1A* in corals will be essential to directly test this hypothesis. Additionally, although our analyses provide insights into host responses associated with probiotic treatment, the in situ transcriptional activity of *E. acroporae* remains unresolved. Future approaches, including rRNA-depletion-based dual RNA-seq or metatranscriptomics, as well as spatially resolved transcriptomics [64] combined with FISH-based localization of CAMAs, may help resolve the functional activity of *E. acroporae* within coral tissues.

### Host flexibility and prospects for field deployment

*Endozoicomonas acroporae* spans a broad geographic range and associates with diverse coral hosts [21], including *S. pistillata* populations in Taiwan [23, 24]. Successful establishment of the same bacterial species in juvenile *Acropora kenti* from Australia through manual inoculation further highlights its host flexibility [38]. Although thermal tolerance was not assessed in treated *A. kenti*, these findings suggest a potential cross-species and cross-regional role for this coral probiotic, which will require validation using a unified experimental framework. Despite these promising outcomes, the in situ performance of *E. acroporae* Acr-14^T^ remains unverified. Field-based validation is essential, yet few coral probiotic studies to date have progressed to this stage. For example, one trial demonstrated that probiotic treatment can halt the progression of SCTLD [65], whereas another reported no detectable off-target effects after three months of continuous application [66], supporting the feasibility of in situ deployment. However, *Endozoicomonas* abundance has been reported to decline during bleaching events [23, 47], suggesting that this symbiosis may be destabilized under thermal stress. Consequently, strategies such as repeated inoculation during heat events may be necessary to sustain the efficacy of *E. acroporae* Acr-14^T^ in natural reef environments.

Even though the responses observed in this study were assessed under *ex situ* conditions, they may reflect functional traits relevant to native *Endozoicomonas*–coral associations, including modulation of host gene expression, oxidative stress mitigation, and microbiome restructuring. Naturally occurring *Endozoicomonas* populations may also contribute additional context-dependent activities, and different *Endozoicomonas* species may play distinct roles in processes such as phosphate cycling [39], sulfur cycling [21], and steroid hormone metabolism [67]. The *Endozoicomonas*–coral re-introduction model established here therefore provides a tractable framework for dissecting these activities and clarifying the broader ecological roles of *Endozoicomonas* in coral holobionts. Field-based validation will be needed to determine whether these responses occur under natural reef conditions.

## Conclusion

In conclusion, this study establishes *E. acroporae* Acr-14^T^ as a coral probiotic and characterizes its capacity to enhance thermal resilience in *S. pistillata*. We show that *E. acroporae*–associated protection is linked to multiple processes, including microbiome restructuring, reduced apoptotic signaling and protein-folding stress signatures, enhanced antioxidant capacity, and altered expression of genes involved in pro-survival signaling and SAMe metabolism. These coordinated responses may help maintain host energy homeostasis and stabilize the coral-algal symbiosis under heat stress (Fig. 7H). Overall, our findings provide molecular insights into host–microbe interactions that promote coral resilience under warming conditions.

## Supplementary Material

Supporting_Information_Revised_wrag173

## Data Availability

All the raw data generated in this study have been deposited in NCBI BioProject under accession number PRJNA1248900. The Whole Genome Shotgun project for *S. pistillata* has been deposited at GenBank under the accession JBNOSB000000000. The version described in this paper is JBNOSB010000000. Code used in this study and additional processed data are available on Zenodo: https://doi.org/10.5281/zenodo.15335716 and is publicly available as of the date of publication. Any additional information in this paper is available from the lead contact upon request.
