# Supplementary material for: Endozoicomonas acroporae enhances coral thermal resilience through host–microbe coordination
Source: ISME J. 2026 Jul 1;20(1):wrag173. doi: 10.1093/ismejo/wrag173 (PMC13455614; doi:10.1093/ismejo/wrag173)
Supplement: Supplementary file 1 [file supporting_information_revised_wrag173.pdf]

Supplementary Materials for

Endozoicomonas acroporae enhances coral thermal resilience through host–microbe coordination

Chih-Ying Lu[1,2,3], Yung-Pei Chang[1], Victor M. Piñon-Gonzalez[1], Thomas D. Lewin[1], Kai-Ning Shih[1,2,3], Po-Shun Chuang[1], Naohisa Wada[4], Sheng-Ping Yu[1], Zhao-Rong Yu[1], Yi-Hua Chen[1], Mo Chen[1], Ching-Hsiang Chin[1], Yu-Jing Chiou[5], Yi-Ling Chiu[1], Isabel Jiah-Yih Liao[1], Hsin-Feng Chang[1], Jui-Hung Yen[1], Mei-Yeh Jade Lu[1], Yi-Jyun Luo[1]*, & Sen-Lin Tang[1,2,6,7]*

* Sen-Lin Tang & Yi-Jyun Luo
Email: sltang@as.edu.tw, yjluo@as.edu.tw

This PDF file includes:

- Supplementary Results
- Supplementary Methods
- Supplementary Figures S1 to S16
- Supplementary Table legends
- Supplementary References

Other supporting materials for this manuscript include the following:

- Supplementary Tables S1 to S6

Supplementary Results

Chromosome-level genome of *S. pistillata* reveals deep clade divergence

S. pistillata clade 1 assembly resolved 14 chromosomes based on contact map analysis (Fig. S8B). *S. pistillata* has been subdivided into four clades according to geographic distribution and molecular markers (Fig. S8A) [1], with clade 1 occurring across the Pacific–Australia region and clade 4 restricted to the Red Sea–Persian/Arabian Gulf–Kenya area. We further assessed genomic divergence among clades by performing pairwise whole-genome comparisons of all available *S. pistillata* assemblies, including *Pocillopora verrucosa* genomes from distinct regions as comparative controls.

Within-clade comparisons showed high similarity among *S. pistillata* genomes (average nucleotide identity [ANI] > 97.7%, alignment fraction [AF] > 0.79), consistent with values observed among *P. verrucosa* genomes (ANI > 97.6%, AF > 0.75) (Fig. S8C). In contrast, comparisons between *S. pistillata* clades 1 and 4 revealed substantial divergence, with ANI values below 91% and AF values under 0.57. Consistent with this, transcriptome alignment efficiency decreased markedly when RNA-seq reads were mapped to non-matching clade references, dropping from an average of 89% to 38% in Taiwan samples (Fig. S8D). This highlights the importance of clade-specific genomic resources for transcriptomic analysis. Together, these results confirm that *S. pistillata* clades are deeply divergent at the genomic level and support previous proposals to recognize them as distinct species [1]. Our newly assembled clade 1 genome provides a necessary foundation for examining host gene expression in response to probiotic treatment.

Functional specialization and phylostratigraphic profiles of *S. pistillata* cell types

To support cell type annotation of *S. pistillata* clade 1, we examined the functional signatures of identified marker genes. Secretory potential was evident in several cell types based on the prevalence of secreted protein markers, such as gastrodermis_1, epidermis_1, nematoblast, and gland cell populations (Fig. S14B). In contrast, neuron_5 was enriched for GPCR-related genes (Fig. S14C), whereas multiple neuronal subtypes exhibited ion channel-associated markers (Fig. S14D), consistent with previously defined clade 4 atlas [2].

We also investigated evolutionary origins of cell-type-specific gene expression patterns through phylostratigraphic analysis [3]. Cell types such as germline_sperm, gastrodermis_6, and algal-hosting cells were enriched for ancient markers, whereas nematoblast, epidermis_1, and calicoblast were associated with younger genes (Fig. S14E). Both nematocytes and nematoblasts were enriched for Cnidaria-specific markers, whereas calicoblasts showed enrichment for genes that emerged in the Hexacorallia and Scleractinia (Fig. S14F), echoing findings from the clade 4 cell atlas [2].

Supplementary Methods

Field work

All corals used in this study belong to *Stylophora pistillata* clade 1, and 80 nubbins from five colonies (16 nubbins each) were collected from Kenting, Taiwan (21.930240°N, 120.745618°E; Fig. S1A) at depths of 7-10 m in June 2023 (seawater temperature: 28°C) by scuba diving. During sampling, only healthy colonies spaced at least 5 m apart were selected to avoid collecting clonal ramets [4]. Nubbins were initially transferred to the National Museum of Marine Biology and Aquarium and acclimated for two weeks in a 5,000 L in-door flow-through tank with natural sunlight and 28°C seawater. For sequencing the *S. pistillata* clade 1 genome, a single colony was collected at 7 m depth from the same site in May 2023. The colony was maintained in a 150-L closed-system aquarium at laboratory prior to processing. Sampling was conducted under permits 1110006783.

Treatment set-up and sampling strategies

Twenty 10-L closed-system aquaria, each consisting of a 2.5-L coral tank and a 7.5-L sump, were employed (Fig. S1B). Water from the sump was circulated to the coral tank using a submersible pump (I-126, ISTA) and returned via a silicone tube, maintaining a flow rate of 3.4 L/min (water turnover time: 44 s). Upon arrival at the laboratory, 80 coral nubbins were allocated evenly among the twenty aquaria, with each aquarium containing four nubbins from different colonies. Four treatments were established, each composing five independent aquaria, making individual aquaria replicates unit (n = 5, true replicate; Fig. S1C). Each aquarium housed nubbins designated for collection at four specific sampling times. At each time point, five nubbins (each from different colonies) from five independent aquaria were collected per treatment.

The four treatments included: (i) ambient temperature with FASW (28°C FASW); (ii) ambient temperature with *E. acroporae* Acr-14^T (28°C *E. acroporae*); (iii) heat stress with FASW (31°C FASW); (iv) heat stress with *E. acroporae* Acr-14^T (31°C *E. acroporae*). All corals underwent an initial two-week acclimation (in total, four weeks of acclimation) at 28°C (ambient temperature of the sampling site). Subsequently, the temperature in heat stress treatments was increased by 0.5°C per day until it reached 31°C, where it was maintained for 12 days, then decreased by 1°C per day to 28°C (Degree Heating Weeks (DHW) [5] = 3.9°C-weeks), and maintained at this temperature for an additional 12-day recovery period. Ambient temperature treatments remained constant at 28°C throughout the experiment (Fig. 1C). The four sampling times occurred at: day 28 (post acclimation, T0); day 42 (early heat stress, T1); day 49 (peak heat stress, T2); and day 63 (post-recovery, T3).

During sampling, nubbins were first rinsed with FASW. Each nubbin was then divided into three (for T0 and T1) or four fragments (for T2 and T3) using a bone cutter, each fragment designated for specific analyses: (i) 16S rRNA gene amplicon sequencing (all time points, n = 5); (ii) antioxidant enzyme activity measurement (all time points, n = 5); (iii) bulk RNA-seq (T1-T3 time points, n = 4); (iv) single-cell RNA-seq (T2 only, n = 1, pooled sample); and (v) fluorescence *in situ* hybridization (FISH, T3 only, n = 2) (Fig. S1C). Fragments intended for DNA extraction were wrapped in aluminum foil, snap-frozen in liquid nitrogen, and stored at -80°C. Those for antioxidant enzyme activity measurements were placed in zip-loc bags and kept on ice for further processing. Fragments for RNA extraction were placed in 2-mL cryotubes containing 1 mL TRIzol Reagent solution (ThermoFisher) and glass beads (0.5 mm diameter Glass beads, BioSpec), vortexed briefly, snap-frozen, and stored at -80°C. Fragments for single-cell RNA-seq were dissociated using calcium- and magnesium-free artificial seawater (CMFSW). Fragments for FISH analysis were fixed overnight in 4% paraformaldehyde (PFA, Wako) and subsequently stored in 70% ethanol at 4°C. Additionally, 500 mL of tank water from each treatment aquarium (n = 1) was collected, filtered through a 0.22 μm membrane, and stored at -20°C until DNA extraction.

Temperature manipulation

Coral tank temperatures were maintained using water baths, with temperature monitored and controlled by Apex Neptune AquaControllers (Neptune Systems) connected to heaters (50-W heater, ISTA). A submersible pump (I-126, ISTA) was placed in individual water bath to ensure homogeneous temperature, and an additional heater was placed in each sump. Each coral tank

contained a thermometer (ISTA). All thermometers were calibrated using a calibrated thermometer (Pocket digital thermometer, DGS) prior to experiments. Chillers were not required, as room temperature was consistently maintained at 23°C.

Culturing conditions and tank maintenance

Artificial seawater (Red Sea Salt, Red Sea) was used in the coral bleaching experiment. An artificial 12:12-hour light:dark cycle was established using LED aquarium lights (Illumagic). Light intensity was set to 150 μmol photons·m^{-2}·s^{-1} during sunrise and sunset and gradually increased to 200 μmol photons·m^{-2}·s^{-1} at midday, measured with an underwater quantum meter (MQ-510, Apogee). Freshly hatched *Artemia* (brine shrimp eggs, O.S.I.) were fed weekly to coral nubbins at a concentration of 20 individuals/mL for two hours after sunset (Fig. S2), following recommended feeding frequencies to meet the nutritional needs of corals during bleaching experiments [4].

Water parameters, including salinity (measured with a refractometer, ATAGO), ammonium, nitrate, phosphate, carbonate hardness, and calcium (measured using Salifert Test Kits) were regularly monitored (Table S1B). When required, parameters were adjusted with deionized water or sodium bicarbonate (Shimakyu) through gentle dosing. Water changes were conducted daily, replacing 20% (2 L) aquarium water. Aquarium air pumps (HX-700, HEXA) provided gentle aeration and water flow in each coral tank.

Probiotic treatment preparation and inoculation

E. acroporae Acr-14^T was originally isolated from *Acropora* sp. in Kenting, Taiwan [6]. Bacteria were cultured at 26°C with shaking at 200 rpm in Modified Marine Broth Version 4 (MMBV4) medium [7], supplemented with an additional carbon source (0.01% glucose). To prepare probiotic treatments, bacterial glycerol stocks were first revived, followed by an activation subculture and a final enrichment subculture in large volume. Cultures were harvested during exponential growth (optical density at 600 nm [OD_{600}] = 0.73 ± 0.19, Table S1A), as determined from growth curves.

On inoculation day, bacterial cultures were centrifuged at 3,000 × *g* for 10 min at room temperature (RT) using 500-mL centrifuge bottles (Polypropylene Bottle, Beckmen). Supernatants were discarded, and cell pellets were resuspended in FASW to prepare concentrated probiotic solutions. Before probiotic addition, all submersible pumps in the sump were turned off to halt water circulation. Probiotic solution was then added to each coral tank to achieve a final OD_{600} of 0.1 (Table S1A), corresponding to 1.19 ± 0.64 × 10^7 viable cells/mL for *E. acroporae* Acr-14^T, as determined by colony-forming unit counts on agar plates. The same volume of FASW was added to control treatments as a placebo. After a 2-hour inoculation, coral nubbins were rinsed twice with FASW and transferred to clean coral tanks pre-filled with temperature-adjusted fresh ASW (Fig. S2). Probiotic inoculations were conducted twice weekly, ensuring no overlap with sampling or feeding events.

Monitoring coral bleaching responses

Pulse-amplitude-modulated (PAM) fluorometry (junior-PAM system, Walz GmbH) was used to measure the maximum quantum yield of photosystem II (Fv/Fm) in coral nubbins, with settings as follows: measuring light intensity = 6; gain = 1; saturation pulse intensity = 8; saturation pulse width = 0.6 s; damping = 2. After a 30-min dark acclimation period, three measurements were randomly taken per nubbin and averaged to obtain representative values. Measurements were consistently taken from the same nubbins throughout the experimental period. Differences in photosynthetic efficiency between treatments were analyzed by one-way ANOVA followed by Tukey's post hoc test (Table S1C).

At each sampling time point, collected nubbins were photographed in a 40 × 40 × 40 (cm) studio (DEEP) with two fixed light sources (5,500 K, LED), using a TG5 digital camera (OLYMPUS) at consistent camera settings. Nubbin coloration was assessed using the Coral Health Chart [8] on a scale from D1 to D6. Differences in color scores between treatments were evaluated with paired-sample t-tests.

Antioxidant enzyme activities

Sample processing

Coral tissues were airbrushed from the collected fragments using ice-cold lysis buffer (50 mM phosphate, 0.1 mM EDTA, 10% [v/v] glycerol, pH 7.0). Samples were centrifuged at 2,000 × *g* for 5 min at 4°C to pellet Symbiodiniaceae cells. Supernatants used for coral host tissue were transferred to new Falcon tubes, centrifuged at 16,000 × *g* for 5 min at 4°C to remove cell debris, aliquoted, snap-frozen, and stored at -80°C until analysis. Symbiodiniaceae cell pellets were resuspended in lysis buffer and washed four times by centrifugation (2,000 × *g*, 5 min, 4°C). Cells were lysed using a bead mill (PowerLyzer 24 Homogenizer, Qiagen) in cryotubes containing 250 mg glass beads (0.5 mm, Biospec) at 3,000 rpm for 15 min, with cycles of 15 s beating followed by 30 min chilling on ice. After centrifugation at 16,000 × *g* for 5 min at 4°C, supernatants were aliquoted, snap-frozen, and stored at -80°C until analysis. We omitted one sample from the 28°C FASW treatment at T3 because the material was insufficient.

Antioxidant-enzyme-activity measurements

CAT activity was measured by mixing 10 µL of sample lysate with 740 µL of potassium phosphate buffer (50 mM, 0.1 mM EDTA, pH 7.0) in a quartz cuvette. The reaction was initiated by adding 50 µL of 320 mM H_2O_2 (final concentration of 20 mM) and monitored at 240 nm for 3 min at RT using a spectrophotometer (U-3900, Hitachi). Measurements were performed in triplicate, and enzyme activity was calculated using an extinction coefficient of 43.6 $M^{-1}cm^{-1}$ [9].

SOD activity was measured using an SOD determination kit (Sigma-Aldrich), following the manufacturer's protocol. A standard curve was generated for each assay using serial dilutions of an SOD standard (Superoxide dismutase, Sigma-Aldrich). Samples were analyzed in triplicate in a 96-well plate, and absorbance was recorded at 450 nm using a microplate reader.

Total protein concentration for both coral hosts and Symbiodiniaceae samples were determined using a Pierce BCA Protein Assay Kit (Thermo Scientific) following the manufacturer's protocol. CAT and SOD activities were normalized against the control treatment at each time point (28°C FASW). Statistical differences in enzyme activities among treatments were assessed using one-way ANOVA followed by Tukey's post hoc tests.

16S rRNA gene sequencing

DNA extraction

Frozen coral fragments were thawed on ice, and tissue samples were collected using an airbrush filled with ice-cold 0.22 µm filtered TE buffer (1 mM EDTA, 10 mM Tris-HCl, pH 8.0). The resulting tissue slurry was transferred into 15-mL Falcon tubes and centrifuged at 12,000 × *g* for 10 min at 4°C to remove the supernatant. Genomic DNA from tissue pellets (or 0.22-µm membranes; tank water samples) was extracted using a DNeasy PowerSoil Pro Kit (QIAGEN) following the manufacturer's protocol. DNA quality was verified by agarose gel electrophoresis and quantified using a NanoDrop 1000 spectrophotometer (ThermoFisher).

Short-read 16S rRNA gene sequencing

16S rRNA gene amplicon sequencing was conducted for collected samples (79 coral and 16 tank water samples). Additionally, six blank samples (DNA extracted from TE buffer) were included to assess potential contamination. The bacterial V6-V8 hypervariable region of the 16S rRNA gene was amplified using the universal primers 968F (5′- AACGCGAAGAACCTTAC-3′)[10] and 1391R (5′-ACGGGCGGTGWGTRC-3′).[11] Each PCR reaction was performed in a total volume of 100 µL containing 1.5 U TaKaRa *Ex Taq* (TaKaRa Bio), 1 x TaKaRa *Ex Taq* buffer, 0.2 mM dNTP mix, 0.2 µM each of forward and reverse primers, template DNA, and nuclease-free water. PCR conditions were the same as a previous work [12], which consisted of an initial denaturation at 94°C for 5 min, followed by 30 cycles of denaturation at 94°C for 30 s, annealing at 52°C for 20 s, extension at 72°C for 45 s, and a final extension step at 72°C for 10 min. PCR products were verified by 1.5% agarose gel electrophoresis, and the target amplicon (424 bp) was excised and purified using the QIAGEN II gel extraction kit (QIAGEN). Barcode-tagging PCR (five cycles) was conducted and DNA was quantified using a NanoDrop 1000 (ThermoFisher) and Qubit HS DNA kit (Qubit Fluorometer, Invitrogen). Samples were pooled in equal quantities, further purified using the QUAquick PCR purification kit (QIAGEN), and sequenced using an Illumina MiSeq platform (300 bp paired-end chemistry) by Yourgene Biosciences (Taiwan).

long-read 16S rRNA gene sequencing

DNA samples, identical to those used for short-read 16S rRNA gene sequencing, were sent to Blossom Biotechnologies (Taiwan) for PCR amplification, library preparation, and sequencing. The protocol followed PacBio guidelines with minor modifications in PCR cycle number and annealing temperature. Briefly, full-length 16S rRNA genes were amplified using the universal primers 27F (5′- AGRGTTYGATYMTGGCTCAG-3′) and 1492R (5′-RGYTACCTTGTTACGACTT-3′) [13], each tagged with unique PacBio barcodes. PCR was performed using KAPA HiFi HotStart ReadyMix (KAPA Biosystems), under the following conditions: initial denaturation at 95°C for 3 min; 30 cycles of denaturation at 95°C for 30 s, annealing at 55°C for 30 s, extension at 72°C for 60 s. Amplified DNA concentration and size were assessed using the Qubit HS DNA kit (Qubit Fluorometer, Invitrogen) and Fragment Analyzer (Agilent Technologies). Amplicons were pooled in equal concentrations, libraries were prepared using the SMRTbell prep kit 3.0, and sequencing was performed on the PacBio Sequel IIe platform.

Fluorescence *in situ* hybridization (FISH)

Testing probe specificity

The Endo-Group B probe [14] (Table S5B) was used to detect *Endozoicomonas* signals in samples. Probe specificity to *E. acroporae* Acr-14^T was verified using the web-based tool TestProbe3.0 (https://www.arb-silva.de/search/testprobe/) with the Silva SSU r138.2 database. Additionally, the specificity of the Endo-Group B probe was experimentally validated with three bacterial cultures: *E. acroporae* Acr-14^T (positive control), *Vibrio coralliilyticus* YB strain, and *Pseudoalteromonas piratica* (both negative controls). Cultures were grown in MMBV4 medium, fixed with 4% PFA (Wako), and individually applied to microscope slides, which were then dried completely at 40°C.

QuickHCR-FISH was performed according to a previously described method [15]. Slides were initially rinsed with 20 mM Tris-HCl (pH 8.0) for 10 min and permeabilized with 1 µg/mL proteinase K in 20 mM Tris-HCl at 37°C for 5 min. Slides were rinsed again with 20 mM Tris-HCl (pH 8.0) for 10 min and prehybridized in hybridization buffer (20 mM Tris-HCl [pH 8.0], 0.9M NaCl, 0.01% SDS, 10% dextran sulfate, 1% blocking reagent, and 25% formamide) for 5 min. For hybridization, cells were incubated first with Non338-initiatorC probe [16] at 46°C for 1 h, followed by incubation with EUB338mix-initiatorH [17] and Endo-Group B-initiatorR probes at 46°C for 2 h. Cells were washed using washing buffer (20 mM Tris-HCl [pH 8.0], 0.149M NaCl, 0.5mM EDTA, and 0.01% SDS).

For signal amplification, Cy3-labeled H1 and H2 amplifier probes, Cy5-labeled R1 and R2 amplifier probes, and Alexa 488-labeled C1 and C2 amplifier probes were individually prepared in amplification buffer (50 mM Na2HPO4, 0.9 M NaCl, 0.01% SDS, 10% dextran sulfate, and 1% blocking reagent), incubated at 95°C for 1.5 min, cooled at 25°C for 30 min, and mixed. Slides were pre-immersed in amplification buffer for 5 min, and then incubated with mixed amplifier probes at 35°C for 30 min. Slides were subsequently rinsed with PBS at 4°C for 10 min, washed with ddH_2O, dehydrated with 100% ethanol, air dried, mounted in VECTASHIELD Antifade Mounting Medium containing DAPI (Vector Laboratories), covered with coverslips, and stored at 4°C until observation.

FISH on coral samples

Eight coral fragments (two colonies from each treatment, n = 2) were collected at T3. Samples fixed with PFA were decalcified using Morse's solution (10% sodium citrate and 22.5% formic acid), exchanging the solution twice over three days at 4°C. Samples were rinsed twice with 10 mM PBS and stored in 70% ethanol at 4°C. Tissue dehydration was performed using a Scientific Excelsior ES Tissue Processor (Thermo Scientific) and embedded in paraffin. Five microscope slides, each containing three 5-µm serial sections, were prepared per sample (40 slides total) using a Lecia RM2125 RTS microtome (Leica). A 50-µm thickness interval between slides was maintained to observe more tissue regions. Prior to QuickHCR-FISH, tissue sections were dewaxed in Xylene (two cycles of 15 min each), dehydrated in 100% ethanol (two cycles of 5 min each), and air-dried. QuickHCR-FISH was then performed as described above.

Microscope observation

Slides were observed with a 40x objective lens on a fluorescence microscope (Ni-L, Nikon) with NIS-elements AR software. The area of *E. acroporae* Acr-14 signals in tissue sections was calculated by measuring overlapping Cy3 and Cy5 signals (with no Alexa 488) using ImageJ software (version 2.14.0/1.54f). High-resolution images were obtained using a 63x objective lens on a confocal laser scanning microscope (Zeiss LSM 880) with ZEN 2.3 (black edition) software. Excitation/emission wavelengths were as follows: DAPI and autofluorescence (405 nm excitation, 417–500 nm emission), Alexa 488 (488 nm excitation, 501–543 nm emission), Cy3 (561 nm excitation, 565–641 nm emission), and Cy5 (633 nm excitation, 652–735 nm emission). Results from bacterial cultures confirmed Endo-Group B probe specificity, detecting signals exclusively in *E. acroporae* Acr-14^{T} and not in negative control bacteria (Fig. S4D).

RNA extraction and bulk RNA-seq

Coral samples were thawed on ice and homogenized for 1 min at 2,000 rpm using a homogenizer (PowerLyzer 24, Qiagen). Homogenates were centrifuged at 12,000 × *g* for 10 min at 4°C and supernatant was collected. Total RNA extraction was performed following the TRIzol reagent protocol. Extracted RNA was aliquoted and stored at -80°C. Prior to sequencing, RNA quality and quantity were assessed using a NanoDrop 1000 (ThermoFisher) and the Qubit HS RNA kit (Qubit Fluorometer, Invitrogen). RNA integrity was evaluated for all samples using a Fragment Analyzer (Agilent Technologies). In total, 51 libraries were constructed with stranded Poly-A protocol. Libraries were sequenced on the NextSeq 2000 sequencing system (Illumina) with 100 bp paired-end reads.

Dissociation and single-cell RNA-seq

Conducting time-course single-cell experiments in corals presents significant technical challenges due to the absence of protocols for long-term preservation of dissociated single cells. Standard fixation methods are incompatible with droplet-based platforms. To overcome this, we developed a fixation-compatible workflow optimized for 10x Genomics.

Coral cell dissociation

At T2, three coral fragments (approximately 0.5–1 cm each) were collected from each treatment group. Each fragment was immersed in 3 mL of calcium- and magnesium-free seawater supplemented with 2% *N*-acetyl cysteine (NAC-CMFSW) in a 24-well plate for 5 min. This step, designed to reduce mucus and prevent interference with downstream cell dissociation, was repeated once. Fragments were then immersed in CMFSW alone for an additional 5 min (total immersion time: 15 min). Following this pre-treatment, fragments were transferred to a fresh well containing 2 mL of CMFSW, and cell dissociation was performed by gentle pipetting using a 1-mL pipette. All dissociation steps were completed within 30 min per sample. To assess cell viability, an aliquot of the cell suspension was stained with 2 µg/mL propidium iodide, a dye that selectively stains dead cells with compromised plasma membranes. The cell survival rate was examined using a hemocytometer under a fluorescence microscope (Ni-L, Nikon). All samples exhibited high viability, with a mean survival rate of 91 ± 1.15% (mean ± SD).

Acetic acid and methanol (ACME) fixation and storage

Cell suspensions from the three fragments in each treatment were pooled, resulting in one combined suspension derived from three coral colonies. A modified ACME fixation protocol was then applied to each pooled sample [18]. Briefly, 2 mL of the pooled cell suspension was fixed by adding 8 mL of freshly prepared ACME solution (methanol:acetic acid:glycerol:7.5% NAC:PBS at a 15:10:10:2:43 ratio) in a 15-mL Falcon tube. Samples were incubated at room temperature for 20 min on a shaker set at 45 rpm. After fixation, cells were passed through a 40-µm cell strainer (pluriSelect) to remove cell clusters and to ensure a uniform single-cell suspension, before being centrifuged at 1,200 × *g* for 5 min at 4°C. To remove the supernatant without disturbing the pellet, most of the liquid was first aspirated using a 1-mL pipette, followed by a 200-µL pipette for the final volume approaching the cell pellet. The cell pellet was then washed with 10 mL of PBS containing 3% BSA (Sigma-Aldrich). This step is critical for trapping dead cells and debris, thereby improving the overall quality of the suspension. After a second centrifugation under the same conditions, the supernatant was discarded using the same pipetting method, and cells were

resuspended in 1 mL of PBS containing 3% BSA and 10% dimethyl sulfoxide (DMSO). DMSO served as a cryoprotectant to prevent cell damage during freezing. Before storage, each fixed suspension was aliquoted into two Lobind tubes (Eppendorf): one for cell counting and viability assessment, and the other for downstream 10x Genomics single-cell sequencing. All samples were stored at -80°C. All reagents were filtered through 0.22 µm membranes prior to use.

Single-cell library preparation and RNA sequencing

Prior to library preparation, fixed cell suspensions were thawed on ice, and the storage solution was replaced with PBS by centrifugation. An aliquot from each cell suspension was stained with Trypan Blue, and cell counts were performed using an EVOS microscope (Invitrogen) with a 10x objective lens and a C-Chip hemocytometer (NanoEntek). Approximately 10,000 cells from each sample were used for single-cell library preparation with the Chromium Single Cell 3' Reagent Kit v3, following the manufacturer’s protocol. Four libraries, corresponding to the four experimental treatments, were generated and sequenced on an Illumina NextSeq 2000 platform using a P3 flow cell (100-cycle kit, 1.0 Cells configuration).

S. pistillata chromosome-level genome sequencing

Algal symbiont removal

Obtaining uncontaminated, high-molecular-weight DNA from coral tissue is challenging due to the presence of intracellular symbiotic algae. To overcome this, we applied menthol treatment to induce bleaching [19] before DNA extraction, thereby minimizing algal contamination [20]. Briefly, coral nubbins were maintained in a 150-L aquarium system. Menthol treatment was conducted in a separate 2-L tank equipped with an air pump to provide aeration and water circulation. Coral nubbins were exposed to 0.2 mM menthol in ASW for no more than 8 h per day, depending on coral condition, and then returned to the main aquarium for recovery. After one week, these nubbins appeared completely bleached with no detectable Symbiodiniaceae under a stereomicroscope (Leica; Fig. S8B).

DNA extraction and sequencing

To extract total genomic DNA from *S. pistillata*, bleached coral nubbins were rinsed with FASW, cut into 0.5 cm fragments, and incubated in 1-mL chaotropic lysis buffer (4.3 M guanidine thiocyanate, 17 mM *N*-Lauroylsarcosine sodium salt, and 25 mM Tris-HCl) in a 2-mL centrifuge tube. Samples were then shaken at 100 rpm and incubated at 55°C for 2 days. Subsequently, 250 µL of the solution was transferred to a new centrifuge tube, mixed with 250 µL PEB buffer (100 mM Tris-HCl [pH 8.0], 10 mM EDTA, and 0.1% SDS), and then mixed thoroughly with 250 µL Phenol/chloroform/isoamyl alcohol (PCI) solution. After centrifuging at 15,000 × *g* for 10 min, the supernatant was transferred into a new tube, mixed with 500 µL chloroform/isoamyl alcohol, and centrifuged again at 15,000 × *g* for 10 min. This chloroform wash step was repeated once. Approximately 400 µL of supernatant was then collected, mixed with 40 µL 3 M sodium acetate and 280 µL isopropanol, and centrifuged at 15,000 × *g* for 20 min at 4°C. The DNA pellet was washed twice with 1 mL ice-cold 70% ethanol, centrifuged at 15,000 × *g* for 10 min at 4°C, air-dried, and finally dissolved in TE buffer. Extracted DNA was aliquoted and stored at -20°C. DNA quality and concentration were assessed using a NanoDrop 1000 (ThermoFisher) and a Qubit HS DNA kit (Qubit Fluorometer, Invitrogen). A whole-genome library was prepared using the SMRTbell Prep Kit 3.0 and sequenced on a PacBio Sequel IIe platform.

Hi-C sequencing

For Hi-C library preparation, we applied a low-speed centrifuge approach to reduce Symbiodiniaceae contamination in the sample. Briefly, coral cells were dissociated from healthy coral fragments using CMFSW as described above. This suspension was centrifuged at 100 × *g* for 5 min at 4°C, and the supernatant was collected, filtered through a 40-µm cell strainer, and centrifuged again under the same conditions. The resulting supernatant was centrifuged at 1,300 × *g* for 5 min at 4°C to pellet cells, which were then snap-frozen in liquid nitrogen and stored at -80°C. This method reduced the proportion of Symbiodiniaceae from 29.1% to 3.3%, as verified using a hemocytometer. The procedure was repeated until approximately 6×10^7 coral cells were obtained. The Hi-C proximity ligation library was prepared with all collected cells using the

Proximo Hi-C (Animal) Kit (Phase Genomics) following the manufacturer's instructions, and sequencing was performed using a NextSeq 2000 sequencing platform with 150-bp paired-end reads.

S. pistillata chromosome-level genome assembly

Primary assembly

The PacBio Sequel IIe platform yielded 2.1 million HiFi reads, totaling 31.7 Gb of sequencing data, with a mean read length of 18 kb, corresponding to approximately 69-fold genome coverage. Reads were assembled de novo using Hifiasm (v0.19.9-r616) [21-23] without incorporating Hi-C data, which generated 194 scaffolds with a total size of approximately 492.9 Mb and a N50 scaffold length of 26.9 Mb. Primary contigs were extracted by converting the GFA output to FASTA format. To remove residual haplotigs and erroneous duplications, we used the Purge_Dups (v1.2.5) pipeline. Briefly, PacBio HiFi reads were aligned to the Hifiasm primary contigs using minimap2 (v2.28-r1209) [24]. Sequencing coverage was estimated by pbcstat and calcuts, generating coverage cutoffs for downstream analysis. Self-alignment of the assembly was performed, followed by the purge of haplotigs based on depth of coverage and overlap. Purged primary contigs were extracted, resulting in a haplotype-purged assembly comprising 59 scaffolds with a total size of 471.2 Mb.

Hi-C based scaffolding

Hi-C Illumina sequencing yielded 166.7 million read pairs, with 50.3 Gb read bases. Hi-C reads were aligned to the scaffold purged contigs using bwa_mem2 [25] and duplicates were marked with samblaster (v0.1.26) [26]. The resulting BAM file was used as input for YaHS (v1.2a.1) [27], which utilizes Hi-C linkage information to scaffold contigs into chromosome-scale scaffolds. This step produced a draft chromosome-level assembly that was then used to generate Hi-C contact maps.

Assembly curation and final polishing

A Hi-C contact matrix was generated using the Juicer [28] pipeline based on read alignments. Chromosome size information was obtained from the final scaffolds using samtools (v1.21) [29]. The draft assembly and corresponding Hi-C contact map were loaded into Juicebox (v1.11.08) [30] for manual curation. Scaffolding corrections were recorded, and an updated AGP file was generated using Juicer post-processing tools. Following manual curation, the final chromosome-level assembly totaled 471.2 Mb, with 97.15% of the sequence anchored into 14 chromosome-scale pseudomolecules. The scaffold N50 was 31 Mb. Assembly completeness was evaluated using BUSCO (v5.7.1) [31] with the metazoan_odb10 database [32], which identified 96.0% of complete BUSCO genes.

Gene model construction

Gene prediction was performed using BRAKER3 (v3.0.8) [33-45], which integrates GeneMark-ES/SP and AUGUSTUS for ab initio gene prediction. Bulk RNA-seq datasets were trimmed with Trimmomatic (v0.39) [46], and post-trimming read quality was assessed using FastQC (v0.12.1). Cleaned short reads were then mapped to the genome assembly using HISAT2 (v2.2.1) [47]. To account for repetitive elements, RepeatModeler (v2.0.6) [48] was used to construct a de novo repeat library, and the genome was subsequently soft-masked using RepeatMasker (v4.1.5). Additionally, the metazoan_odb10 database [32] was downloaded. The resulting BAM file, soft-masked genome, and metazoan protein database were used as inputs for BRAKER3. The final annotation contained 25,918 genes encoding 28,789 transcripts. The completeness of predicted gene models was evaluated using BUSCO (v5.7.1) [31] with the metazoan_odb10 dataset, yielding a 97.0% BUSCO completeness (single: 84.0%, duplicated: 13.0%, fragmentated: 0.9%, missing: 2.1%).

Microbiome analysis

Short-read 16S data processing

Amplicon sequencing data generated by the Illumina MiSeq platform were processed using QIIME2 (v2021.8.0) [49]. Raw reads were reoriented, primers trimmed, and sequences

demultiplexed using the cutadapt plugin (v3.4) [50]. Forward and reverse reads were truncated at quality score threshold of 25 and denoised using the DADA2 algorithm [51]. Taxonomy of ASVs was assigned using the classifier-consensus-vsearch plugin [52] against the SILVA v138 SSU NR99 reference database [53, 54].

long-read 16S data processing

PacBio HiFi full-length 16S rRNA gene sequencing data were analyzed using a dedicated full-length 16S analysis pipeline implemented in Nextflow (v0.7) [55]. Briefly, HiFi reads with a Q20 quality threshold were trimmed, and primers were oriented using cutadapt [50]. ASVs were obtained by denoising using DADA2 [51] in QIIME2 [49]. Taxonomic classification was performed using VSEARCH against GTDB r207 [56], Silva v138 [53, 54], and RefSeq + RDP [57] databases. No minimum frequency or sample filter was applied, and the minimum sequence length retained was set to 900 bp.

Data filtering and decontamination

ASVs assigned to chloroplast, mitochondria, eukaryota, archaea, or unclassified kingdoms and phyla were removed, as were singleton ASVs. Decontamination was performed by removing ASVs whose averaged reads counts were higher in blanks than in formal samples. In total, 34 ASVs and 37 ASVs were identified as background microbial signals in the short-read and long-read 16S datasets, respectively (Table S2A and B). After filtering and decontamination 2,861 (short-read) and 3,027 (long-read) ASVs remained, with average read counts of 22,154 (short-read) and 6,266 (long-read).

Community profiling

Microbial relative abundance profiles at the class level were visualized using phyloseq [58] and ggplot2 [59]. Long-read sequencing provided species-level resolution and confirmed all *Endozoicomonas* ASVs as *E. acroporae*. Rarefaction curves were generated using the “ggrare” function in phyloseq-extended package. For short-read data, a sample from the 31°C FASW treatment at T1 was excluded from downstream analysis due to low read count (< 1,000 reads). Further analyses were not performed on long-read data due to low sequencing depth (< 1,000 reads for approximately one-third of samples) (Fig. S3D), primarily caused by abundant chloroplast sequences in coral samples.

Alpha diversity metrics, including AVS richness and the Shannon index, were calculated using the microbiome package after rarifying samples to an even depth using the SRS package [60]. Differences between groups were assessed using Wilcoxon rank-sum tests. Multivariate analyses were performed at the ASV level using relative-abundance-transformed data. Bray-Curtis distances between samples were calculated using vegan [61] and visualized with principal coordinates analysis (PCoA). Microbial community differences between treatments were assessed by permutational multivariate analysis of variance (PERMANOVA) with 999 permutations using the “adonis” function. Multivariate dispersion of treatments was evaluated with “betadisper” and tested using ANOVA. Significantly differences between coral and tank water samples were observed in both alpha and beta diversity ($P < 0.05$; Fig. S3E and F).

Differential abundance analysis

Differential abundance analyses at the genus level employed a consensus approach using ANCOM-BC [62] and MaAsLin2 [63] for robust interpretation [64]. The control treatment (ambient temperature with FASW) was used as the reference. ANCOM-BC was executed with parameters: “tax_level” set to genus, “conserve” set to TRUE, and “prv_cut” at 0.05. For MaAsLin2, data were agglomerated at the genus level, transformed to relative abundances, and analyzed using default parameters. Only genera significantly different by both methods ($P < 0.05$) were accepted. Genera with low relative abundance (< 0.1% across all treatments) were excluded (Table S3A-D). Results highlighting genera potentially contributing to probiotic effects were visualized with Venn diagrams and ComplexHeatmap (v2.18.0) [65].

Association network analysis

Microbial association networks at the genus level were constructed using NetCoMi (v1.1.0) [66]. To mitigate data sparsity, the 60 genera with the highest prevalence across samples were retained using the "filtTax" and "filtTaxPar" functions. Data were normalized via centered log-ratio (clr) transformation. Genera associations were measured using SparCC correlations, and filtered with a threshold of 0.3. Clusters were identified through greedy optimization of modularity, and the resulting network was visualized using Gephi (v0.10.1). All analyses for microbiome data were conducted in R (v4.3.2) and colorblind safe color schemes were utilized for figures.

S. pistillata clade 1 and clade 4 genome comparison

Genetic identity based on ITS rDNA

To confirm the genetic identity of these samples, we amplified the ITS1-5.8S-ITS2 region using the anthozoan-universal primer pairs 1S (5′-GGTACCCTTTGTACACACCGCCCGTCGCT-3′) and 2SS (5′-GCTTTGGGCGGCAGTCCCAAGCAACCCGACTC-3′) [67]. PCR conditions were performed as follows: initial denaturation at 95°C for 4 min; 30 cycles of denaturation at 94°C for 30 s, annealing at 55°C for 60 s, extension at 72°C for 2 min; and a final extension at 72°C for 10 min. PCR products were summited to Genomics Biotechnologies (Taiwan) for Sanger sequencing. Additionally, an *S. pistillata* genome recently released in NCBI without geographic origin information (GCA_964205215.1) was included by extracting its ITS sequence. Obtained sequences were aligned with 172 publicly available *S. pistillata* ITS rDNA sequences [1] using MEGA11 [68]. Phylogenetic analysis of the 174 ITS sequences was performed using IQ-TREE (v2.0.6) with ModelFinder enabled [69] and 1,000 bootstrap replicates. The resulting treefile was imported into iTOL (v7.0) [70] to construct a phylogenic tree rooted with an outgroup. As expected, *S. pistillata* samples used in this study clustered with clade 1, whereas the GCA_964205215.1 sequence clustered with clade 4, suggesting its origin from the Red Sea-Persian/Arabian Gulf-Kenya region (Fig. S8A).

Pairwise whole-genome alignments

To investigate genomic conservation and divergence between *S. pistillata* clades 1 and 4, pairwise whole-genome alignments were conducted using genomes from: GCA_964205215.1 (*S. pistillata*, location unknown); GCF_002571385.2 (*S. pistillata*, Red Sea); GCA_032172095.1 (*S. pistillata*, Australia); GCF_036669915.1 (*Pocillopora verrucosa*, Spratly Islands); GCA_014529365.1 (*P. verrucosa*, Red Sea); GCF_030620025.1 (*P. verrucosa*, Australia); and the genome assembled in this study (*S. pistillata*, Taiwan). Genome scaffold lengths were determined using samtools (v1.21) [29]. Scaffolds shorter than 1,000 bp were excluded from the analysis. Pairwise alignments were performed using MUMmer (v4.0.0) [71]. One-to-one alignments were filtered using the delta-filter function, and alignment coordinates were extracted with show-coords. Based on these coordinates, average nucleotide identity (ANI) was calculated as followed:

$$\text{ANI} = \sum(\text{ID\%} * \text{Length of Alignment}) / \sum(\text{Length of Alignment})$$

The alignment fraction (AF) was calculated as:

$$\text{AF} = \sum(\text{Length of Alignment}) / (\text{Length of the Query Genome})$$

These metrics were adapted from previously described methods [72]. Results were visualized in R using the scatterpie package.

Comparative alignment rates

RNA-seq data from *S. pistillata* samples from Taiwan (this study) and the Red Sea [73] were used to compare alignment rate against the clade 1 (this study) and clade 4 (GCA_964205215.1) genomes as references. Reference genome indices were built using HISAT2 (v2.2.1) [47], incorporating exon and splice-site annotations from their respective gene models. Trimmed bulk RNA-seq reads, with or without removal of Symbiodiniaceae sequences (*Cladocopium goreaui* for clade 1, GCA_947184155.2; *Symbiodinium microadriaticum* for clade 4, GCA_001939145.1), were then aligned to each reference genome index.

Bulk RNA-seq data analysis

Mapping reads to host genome

Demultiplexing was performed by the sequencing facility. Adapter sequences and low-quality bases were trimmed using Trimmomatic (v0.39) [46] and checked with FastQC (v0.12.1). We followed recommended best practices for transcriptome analysis, employing highly accurate tools [74]. A genome index for *S. pistillata* clade 1 was built as described above, and trimmed reads were aligned using HISAT2 (v2.2.1) [47]. Unaligned reads were saved for downstream analysis of the Symbiodiniaceae transcriptome. Across 51 samples, alignment rates to the host genome ranged from 58.42% to 92.52%, with bleached samples showing notably higher alignment rates. SAM files were converted to compressed BAM format and sorted using samtools (v1.21) [29].

Mapping reads to the Symbiodiniaceae genome

To identify Symbiodiniaceae lineages present in corals, the *LSU* region was amplified from sample DNA using primers 28S-forward (5′-CCCGCTGAATTTAAGCATATAAGTAAGCGG-3′) and 28S-reverse (5′-GTTAGACTCCTTGGTCCGTGTTTCAAGA-3′) [75] with the following PCR conditions: initial denaturation at 90°C for 2 min, followed by 35 cycles of 94°C for 1 min, 60°C for 1 min, 72°C for 1 min, and a final extension at 72°C for 5 min. PCR products were submitted to Genomics Biotechnologies (Taiwan) for Sanger sequencing. BLAST searches against the NCBI core nucleotide database indicated that all five coral colonies were predominantly colonized by *Cladocopium sp.* (100% query coverage, 99.23% identity). The *Cladocopium goreaui* genome (GCA_947184155.2) was downloaded from NCBI. Read alignment was performed as for the host while using the *C. goreaui* genome and gene models to build the reference index. Using host-unaligned reads, alignment rates ranged from 42.4% to 75.7% across samples; two severely bleached samples showed markedly lower alignment (9.7% and 1.3%; colonies d and e at T3) and were excluded from downstream analyses.

Estimating transcript abundance

Sorted BAM files were assembled using StringTie (v2.2.3) [76], guided by BRAKER-derived gene models. Assemblies were merged into a unified transcriptome annotation using the --merge function, resulting in 41,541 genes identified in the *S. pistillata* transcriptomes. Transcript abundance per sample was estimated using StringTie (v2.2.3) [76], with the *S. pistillata* merged annotation as the reference for host transcripts and the *C. goreaui* gene models as the reference for symbiont transcripts.

Transcriptome functional annotation

Transcript sequences were extracted from the genome using gffread (v0.12.7) based on the merged annotation. Open reading frames (ORFs) were predicted using TransDecoder (v5.7.0) with default parameters. Predicted protein functions were annotated using the following approaches: (i) Pfam domain identification using Pfamscan and the Pfam database (v37.0) [77]; (ii) signal peptide prediction using SignalP (v6.0) [78]; (iii) gene names assignment via DIAMOND aligner (v2.1.10) [79] against the human genome (vGRCh38, Ensembl release 113) with an e-value threshold of 1e-5; (iv) gene ontologies annotation using eggNOG-mapper (v2.1.12) [80] with the eggNOG database (v5.0.2) [81]; and (v) comprehensive transcriptome annotation using Trinotate (v4.0.2) [82]. For the Symbiodiniaceae transcriptome, only methods (iv) and (v) were performed.

Differential expression analysis

Transcript abundance estimates were imported into R with tximport [83], utilizing a tx2gene table linking transcripts to genes. Differential gene expression analysis was performed at the gene level using DESeq2 [84]. Pre-filtering was performed to remove genes with fewer than 10 counts in the smallest group size (n = 3 in the 28°C *E. acroporae* treatment at T2), leaving 30,708 of 41,541 genes for the coral host and 25,130 of 40,390 genes for Symbiodiniaceae. Comparisons were made at each time point against the control (28°C FASW) or directly between the 31°C FASW and 31°C *E. acroporae* treatments. Genes with adjusted $P < 0.05$ were considered differentially expressed (DEGs). Results were visualized with the EnhancedVolcano package.

Expression profiles were explored using principal component analysis (PCA) plots generated from variance-stabilized (*vst*) normalized counts in DESeq2 and visualized with ggplot2 [59]. Upregulated and downregulated DEGs from each comparison were illustrated using

Venn diagrams at each sampling time point, created with the VennDiagram package. Expression levels of selected genes were visualized using ComplexHeatmap (v2.18.0) [65] with data normalized by z-scores.

Functional analysis

Gene Ontology (GO) enrichment analysis was performed using clusterProfiler (enricher function) [85]. GO terms and gene information was prepared from the annotated transcriptome, with all annotated, pre-filtered genes serving as the background. Terms significantly enriched (adjusted P value < 0.05 and P value < 0.0005) were visualized with ggplot2 [59], presenting the top 5 (sort by adjusted P value) biological processes. Terms redundant with highly similarity, e.g., response to topologically incorrect protein vs cellular response to topologically incorrect protein, or irrelevant to coral biology, e.g., wing disc morphogenesis, were excluded.

Gene set enrichment analysis (GSEA) was conducted using clusterProfiler (GSEA function with fgsea algorithm) [85]. Genes were pre-ranked using the Wald statistic from DESeq2 results, and enrichments were considered significant at adjusted P < 0.05. GSEA results were visualized following the same rules those used for GO enrichment analysis, using the ggplot2 and enrichplot packages.

scRNA-seq data analysis

Generating count matrix and data filtering

Demultiplexing was performed by the sequencing facility. Cell Ranger (v8.0.1) [86] was used for sequence alignment using the cellranger count function with –expect-cells set to 10,000 for all four libraries. The reference was generated from the *S. pistillata* clade 1 genome and the StringTie-derived transcriptome annotation with genes lacking strand information removed. For the four libraries, sequences aligned to the host transcriptome captured 8,855, 9,219, 10,015, and 8,626 cells, respectively, corresponding to four treatments: 28°C FASW, 28°C *E. acroporae*, 31°C FASW, and 31°C *E. acroporae*. Median genes per cell were 732, 881, 748, and 715, respectively, while median unique molecular identifiers (UMIs) per cell were 1,738, 2,104, 1,699, and 1,839. Sequencing saturation ranged from 72% to 78.5%.

CellBender (v0.3.2) [87] was used to mitigate background noise in the count matrices using parameters: -expected-cells 10,000, -total-droplets-included 30,000, -fpr 0.01, and -epochs 150. Additional parameters adjusted according to the ELBO learning curve included -learning-rate (2e-5 to 1e-5) and -low-count-threshold (set at 100 or disabled). Noise-removed matrices were imported into Seurat (v5.1.0) [88] using scCustomize (v3.0.1). Cells with low (< 100 UMIs) or very high (> 8,000 UMIs) molecule counts were removed. Potential doublets were removed using scDblFinder (v1.16.0) [89]. After filtering, an average of 7,409 cells remained per sample, with a median of 684 genes and 1,488 UMIs per cell, comparable to previous stony coral single-cell studies [2].

Cell clustering and cell type annotation

After filtering, the four Seurat objects were merged, normalized with the NormalizeData function, and subset to the 2,000 most variable features using the FindVariableFeatures function. Data were scaled, and PCA dimensional reduction was performed. Dimensionality for clustering analysis was determined with an elbow plot, selecting the first 30 principal components. Clusters were identified using FindNeighbors and FindClusters with a resolution of 0.6. Clusters were visualized via UMAP (DimPlot function). Marker genes defining clusters were identified with FindAllMarkers (parameters: min.diff.pct = 0.1, only.pos = TRUE). Violin plots (VlnPlot) and heatmaps (ComplexHeatmap) were generated to visualize marker gene expression. Differences in cluster proportions between treatments were tested and visualized using scProportionTest [90].

To annotate cluster identity, 491 highly variable genes from a published single-cell data set of *S. pistillata* (clade 4) [2] were aligned against single-cell markers of *S. pistillata* (clade 1) using blastn (v2.16.0) [91]. Reciprocal best hits (RBH) identified orthologous genes with thresholds: percent identity ≥ 85%, e-value ≤ 1e-5 (bidirectional), and query coverage ≥ 50% (at least one direction; Table S5A). A total of 280 orthologous marker genes were identified, and 181 highly variable markers (average $\log_2$ fold-change > 1.7) were used to classify cell clusters. Visualization of results was performed using ggbump package.

Gene age estimation

For each gene in the *S. pistillata* clade 1 genome in BRAKER-derived gene models, a phylostratigraphic age [3, 92] was estimated using GenEra (v1.2.0) [93]. GenEra uses DIAMOND [79] searches of the NR database to assign genes to one of 12 categorical age classes (or phylostrata), from youngest (rank 12, *S. pistillata*-specific) to oldest (rank 1, found in all cellular organisms). These numerical ranks were used to calculate the average age of marker genes for each single cell cluster. The $\log_2$ fold change in the proportion of marker genes of each age versus the proportion of all genes of each age was calculated as described previously [2]. Data were presented as heatmaps plotted in R with ComplexHeatmap (v2.18.0) [65]. Benjamini-Hochberg-corrected Fisher's exact tests were applied to test whether the set of marker genes for each cluster is enriched or depleted for genes from each phylostratum.

Differential expression and functional analysis

Differential expression analysis was performed at the single-cell level using Seurat (v5.1.0) [88] FindMarkers function with the MAST algorithm. Differential expression was tested between the control (28°C FASW) and each of the other three treatments in the same cell type. Genes with an adjusted $P < 0.05$ were considered significantly differentially expressed. GO enrichment analysis was performed as described above. Significantly enriched GO terms related to coral heat stress responses were visualized using ggplot2. Intersection analysis of significantly expressed genes identified in bulk and single-cell RNA-seq data was visualized using UpSetR [94] package. Expression levels of key genes were visualized on UMAP plots using the Seurat (v5.1.0) [88] FeaturePlot function.

Statistical analysis

All statistical analyses were performed in R (v4.3.2) using RStudio (v2023.12.1+402). Specific statistical tests used are indicated in each method section or figure legend. Data normality and homogeneity of variance were assessed using Shapiro-Wilk and Levene's tests, respectively. Sample sizes or gene numbers for each analysis are indicated by "n" in corresponding figure panels or legends.

Supplementary Figures

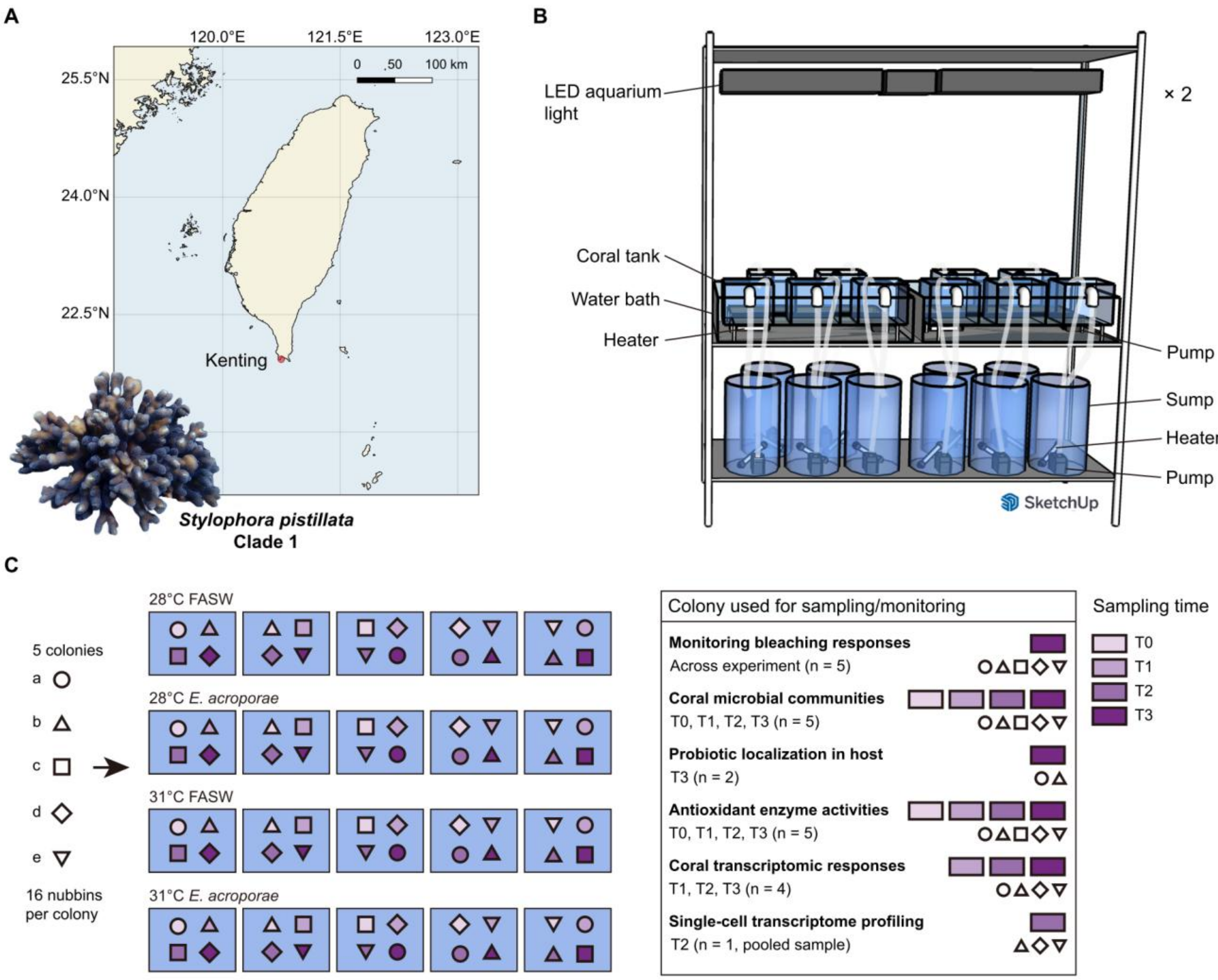

Figure S1. Schematic overview of experimental tank configurations and replication design. (**A**) Sampling map and photograph of *Stylophora pistillata* clade 1. (**B**) Schematic of the experimental tank used in the coral bleaching experiment. (**C**) Replication design for each assay. Individual coral colonies are represented by distinct shapes, with colony codes labeled on the left. Shape and fill color indicate the inclusion of colonies in different assays.

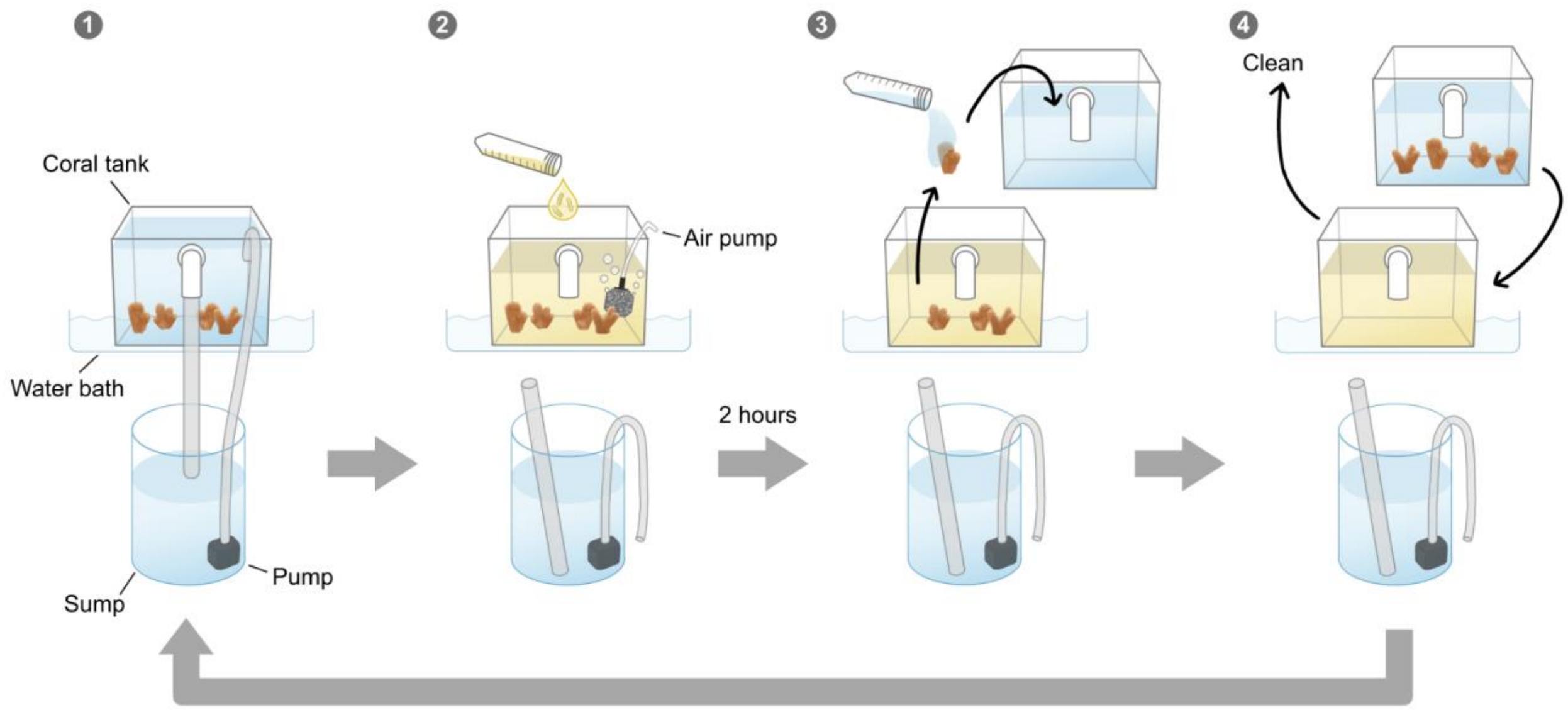

Figure S2. Detailed probiotic inoculation and feeding procedures. Step 1: Water circulation between the coral tank and its sump was halted by turning off the submersible pump and disconnecting silicone tubing. Step 2: Concentrated probiotic solutions or placebo (FASW) were added to the coral tank, with water movement maintained by an air pump during inoculation. Step 3: After 2 h of inoculation, coral nubbins were rinsed with FASW and transferred to a new coral tank prefilled with temperature-adjusted artificial seawater. Step 4: After all coral nubbins were transferred, the used coral tank was replaced and cleaned. Silicone tubing was then reconnected, and the submersible pump was reactivated to resume water circulation with the sump. Feeding was conducted following the same procedure as probiotic inoculation, with the probiotic solution replaced by *Artemia*.

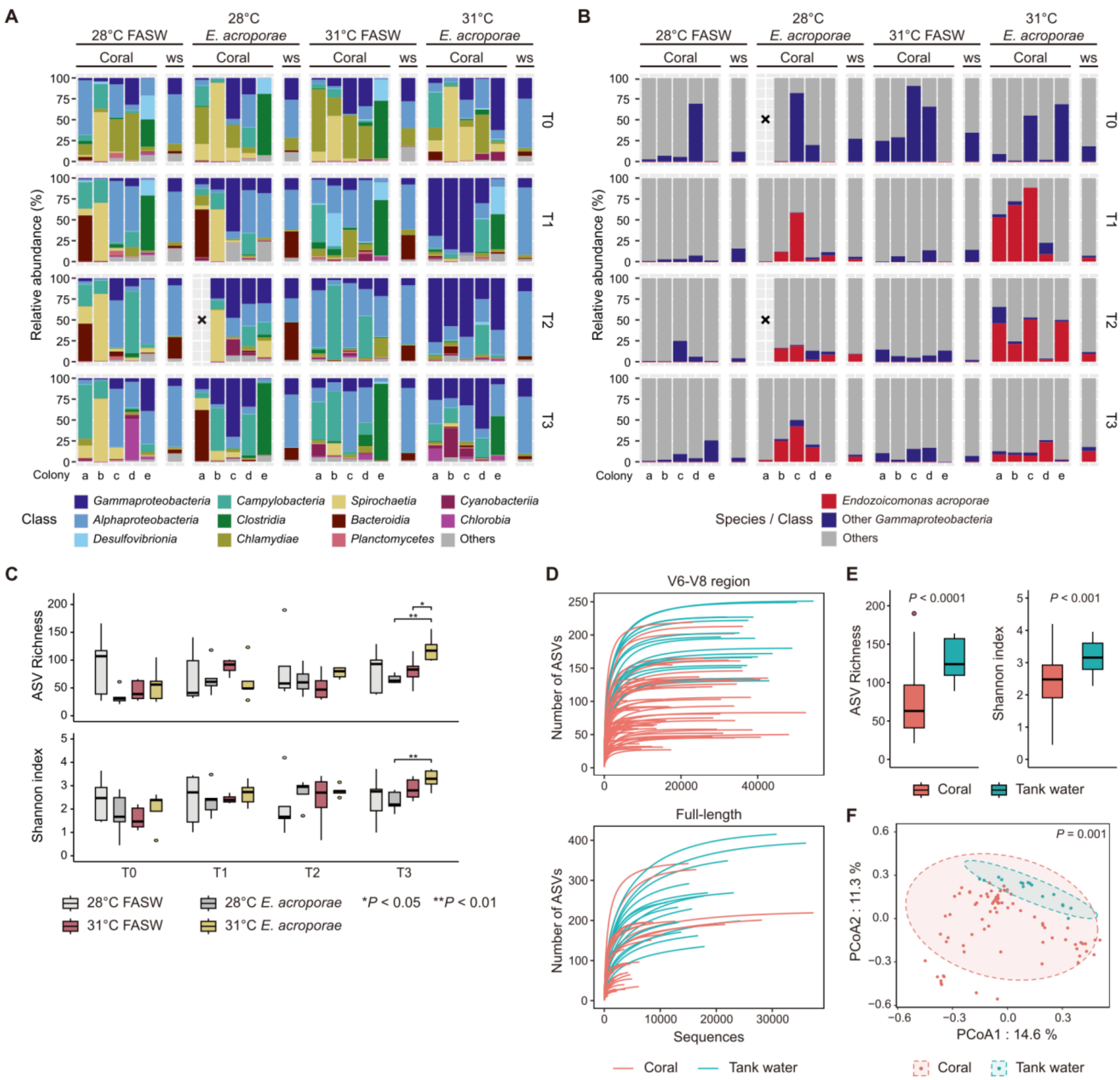

Figure S3. Microbial community composition and sequencing quality assessment. (**A**) Relative abundance of bacterial classes across treatments, determined by short-read 16S rRNA gene amplicon sequencing. Tank water samples, ws; lost sample, x. (**B**) Relative abundance of *E. acroporae* and other *Gammaproteobacteria* in samples, determined by long-read 16S rRNA gene amplicon sequencing. All ASVs assigned to the genus *Endozoicomonas* were classified as *E. acroporae*. Lost or failed-to-sequence samples, x. (**C**) ASV richness and Shannon index of coral samples across treatments (n = 5; Wilcoxon signed-rank test; **P* < 0.05; ***P* < 0.01). (**D**) Rarefaction curves for all samples based on short-read and long-read 16S rRNA gene amplicon sequencing. (**E**) ASV richness and Shannon index comparing coral (n = 78) and tank water (n = 16) samples (Wilcoxon rank-sum test). (**F**) PCoA illustrating microbial community differences between coral (n = 78) and tank water (n = 16) samples. Ellipses represent 95% confidence intervals around group centroids (PERMANOVA).

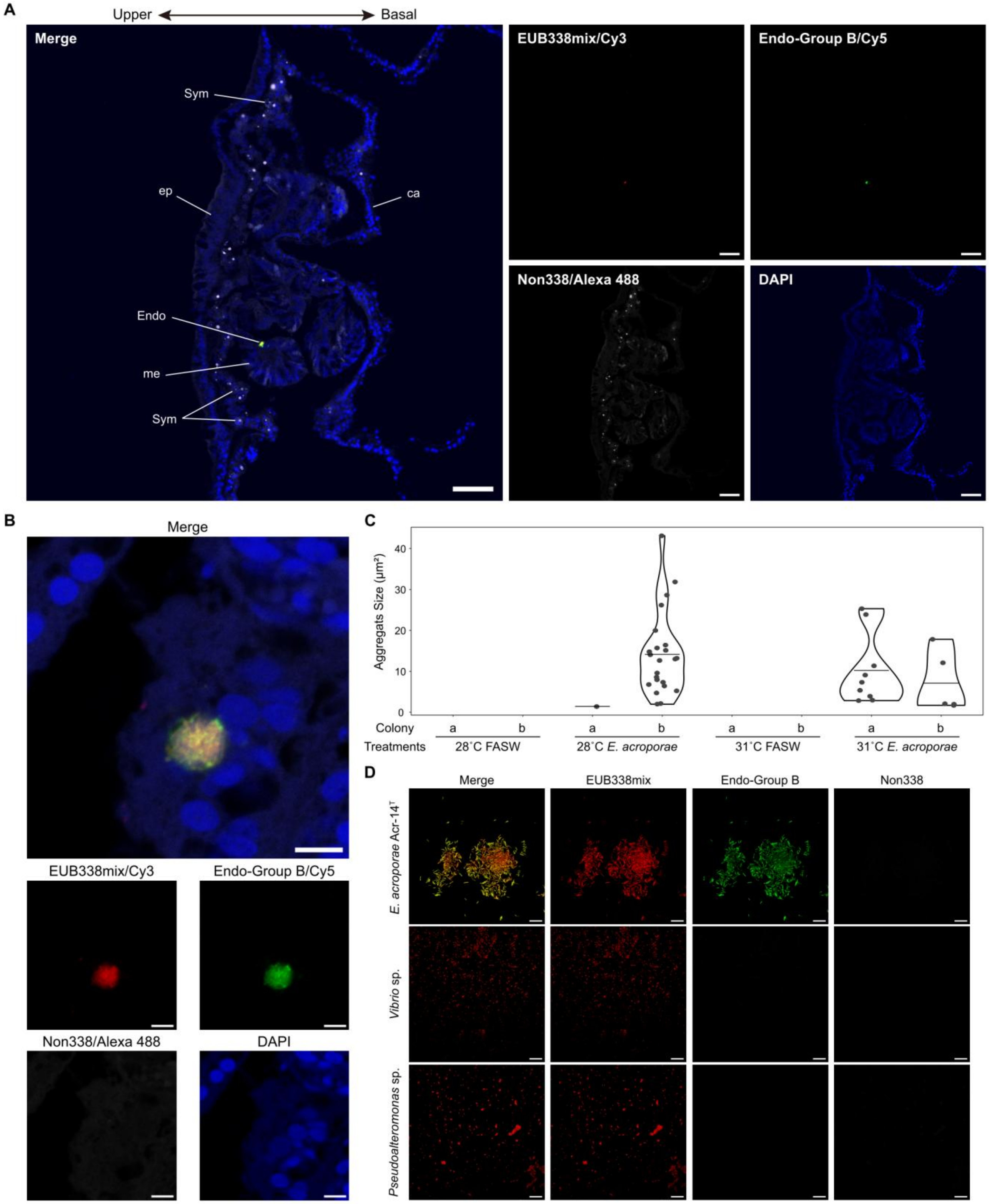

Figure S4. ***E. acroporae*** **forms coral-associated microbial aggregates (CAMAs) in probiotic-treated coral tissues.** (**A**) Representative fluorescence *in situ* hybridization (FISH) images (split and merged channels) of a coral tissue section from the 31°C *E. acroporae*

treatment, showing *Endozoicomonas* signals within mesenterial filaments. ca, calicoblastic cell layer; Endo, *Endozoicomonas*; ep, epidermal layer; me, mesenterial filaments; Sym, Symbiodiniaceae. Scale bars, 50 µm. (**B**) High-magnification view of *Endozoicomonas* signals (split and merged channels) in a sample from the 28°C *E. acroporae* treatment. Scale bars, 5 µm. (**C**) Quantification of *Endozoicomonas* aggregation size across the four treatments (n = 30 tissue sections per treatment). (**D**) Validation of FISH probe specificity using the universal prokaryotic probe EUB338mix and the Endo-Group B probe with three bacterial cultures: *E. acroporae* Acr-14^T, *Vibrio coralliilyticus* YB strain, and *Pseudoalteromonas piratica.* Scale bars, 10 µm.

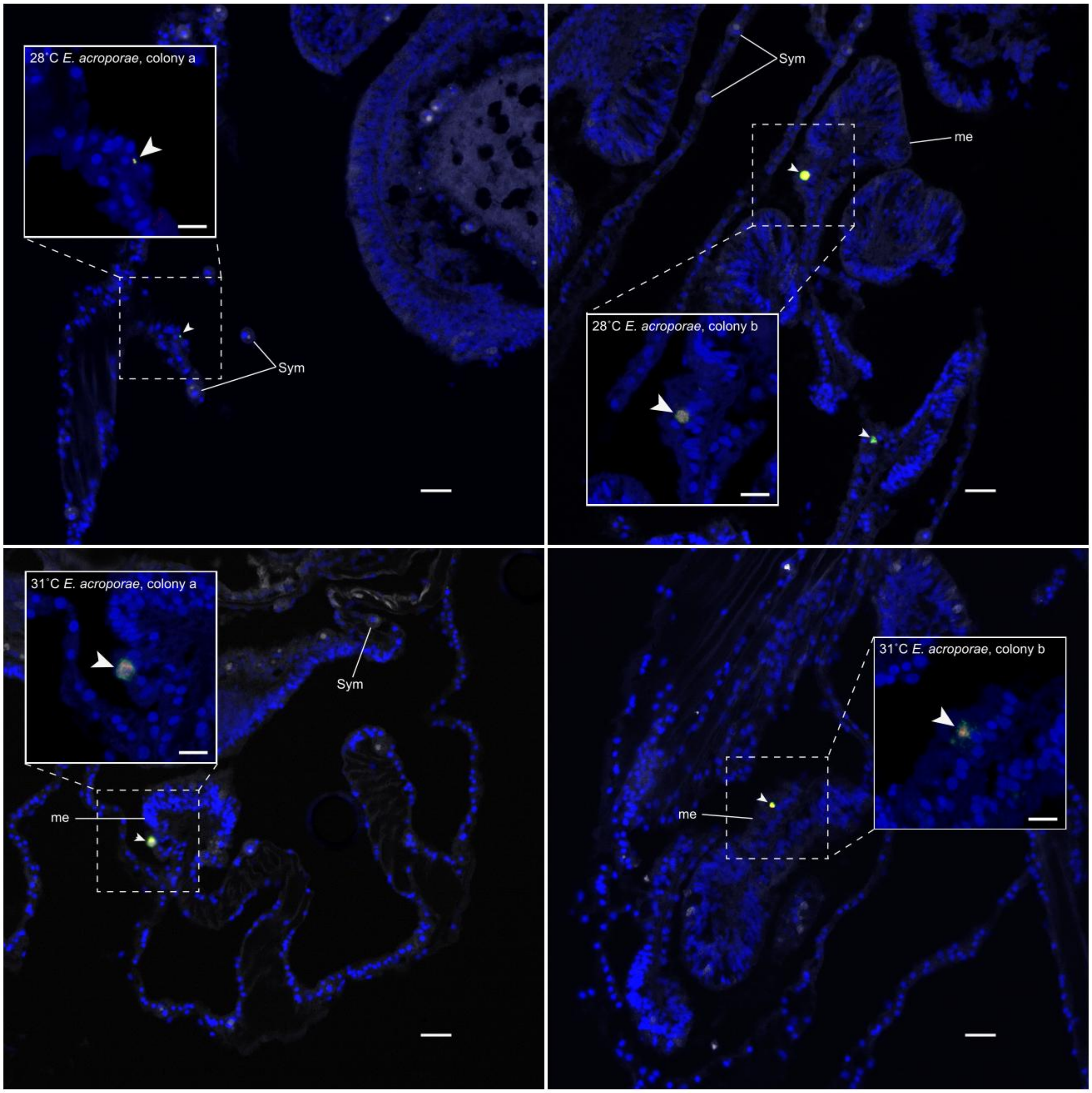

Figure S5. Zoomed-out views of representative FISH images displayed in Fig. 2. Broader tissue-section views of the representative FISH images (merged channels) shown in Fig. 2, captured with a Nikon fluorescence microscope. White arrows indicate *Endozoicomonas* signals. Sym, Symbiodiniaceae; me, mesenterial filaments. Scale bars: zoomed-in image, 10 μm; zoomed-out image, 20 μm.

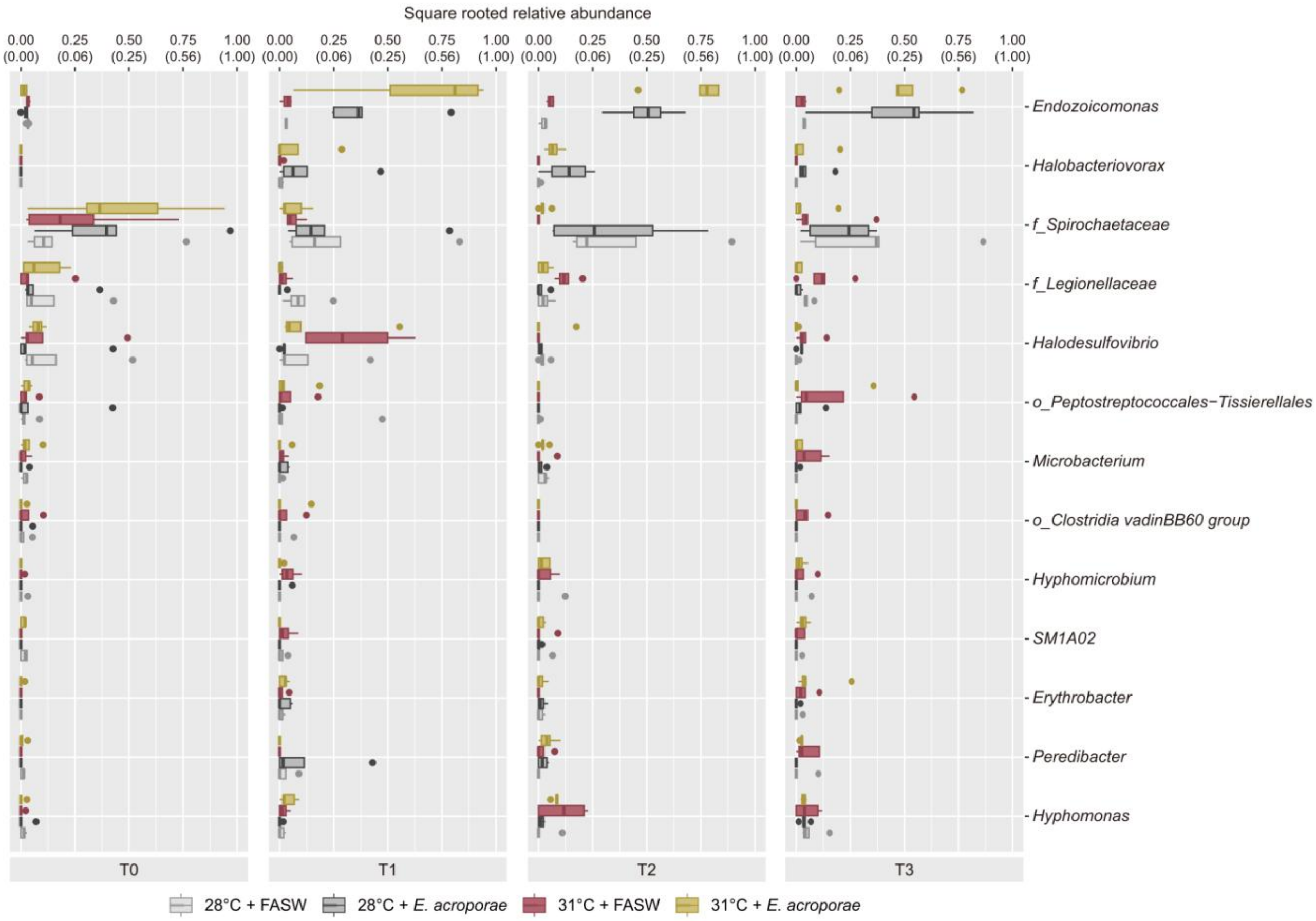

Figure S6. Square-rooted relative abundances of differentially abundant genera. Square-rooted relative abundances of genera of particular interest identified by differential abundance analyses. Untransformed relative abundances are provided in parentheses on the x-axis.

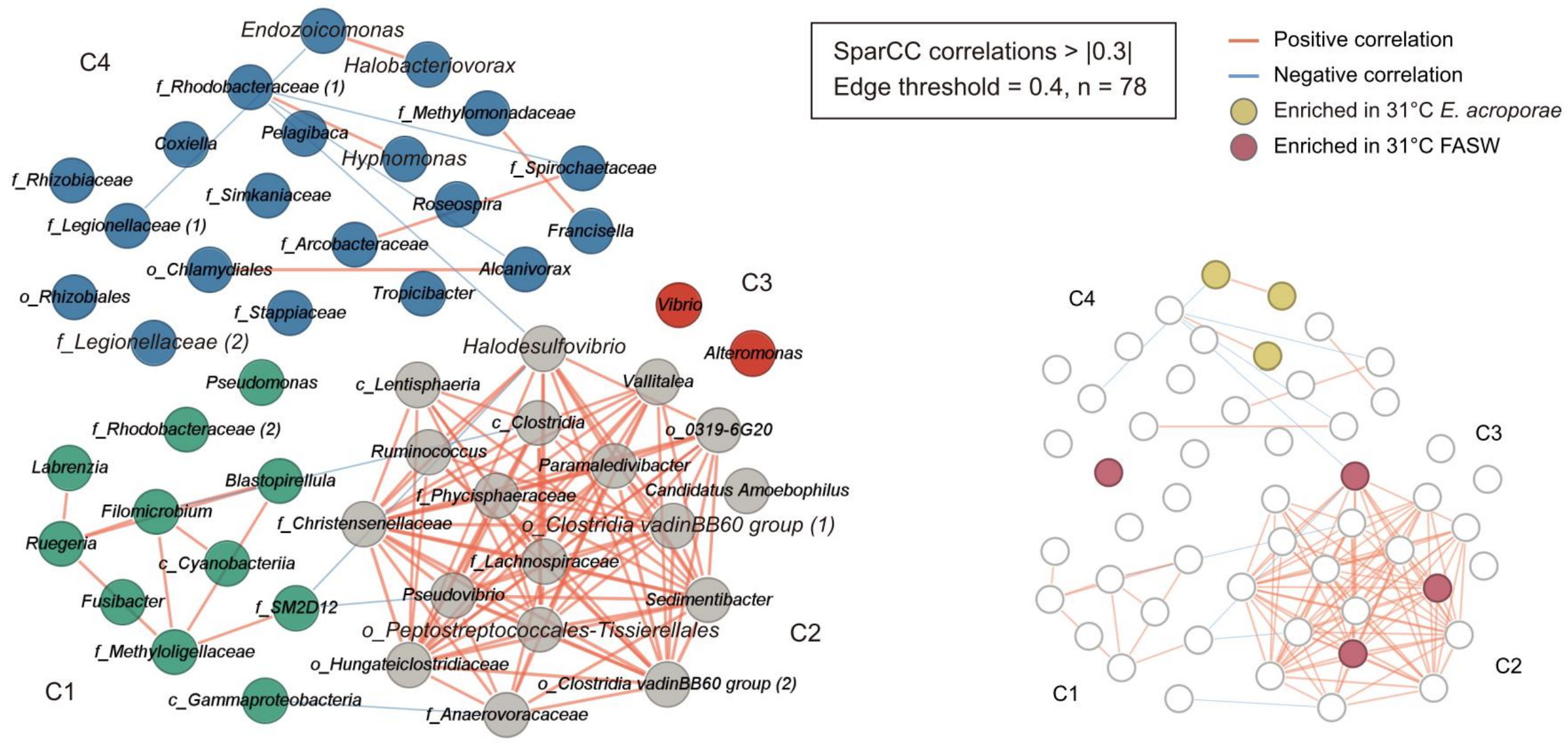

Figure S7. Microbial co-occurrence network of coral samples. SparCC correlation networks of the 60 most prevalent bacterial genera in coral samples. Small panels show networks overlaid with genera specifically enriched in the 31°C FASW or 31°C *E. acroporae* treatments. Network clusters are labeled with "C" followed by a number.

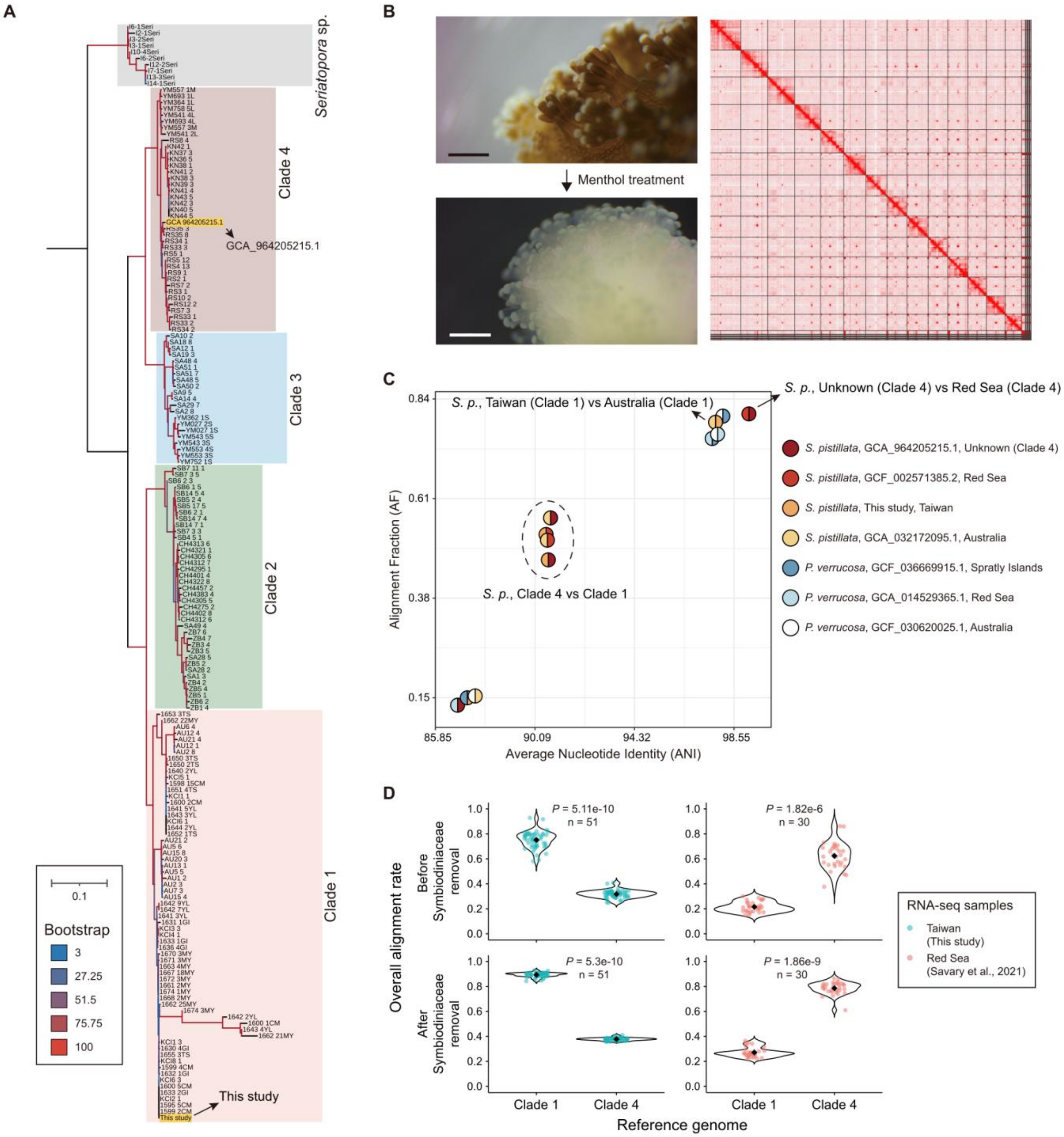

Figure S8. Extensive genomic divergence between *S. pistillata* clades 1 and 4. (**A**) Phylogenetic tree of *S. pistillata* ITS rDNA sequences obtained from this study, a publicly released genome (GCA_964205215.1), and a previous study [1]. The tree is rooted using an outgroup (*Seriatopora* sp.). (**B**) Hi-C contact map of the chromosome-level genome assembly of *S. pistillata* clade 1 and photographs illustrating menthol-induced coral bleaching (left). Scale bars, 1 mm. (**C**) Pairwise whole-genome alignments illustrating genetic divergence between *S. pistillata* clades 1 and 4 genomes. Colors in the pie chart indicate genomes used for alignment. (**D**) Overall alignment rates of RNA-seq reads mapped to clade 1 or clade 4 *S. pistillata* reference

genomes, with (bottom) or without (top) Symbiodiniaceae read removal. Each point represents one sample, and black diamonds indicate group means (Wilcoxon signed-rank tests).

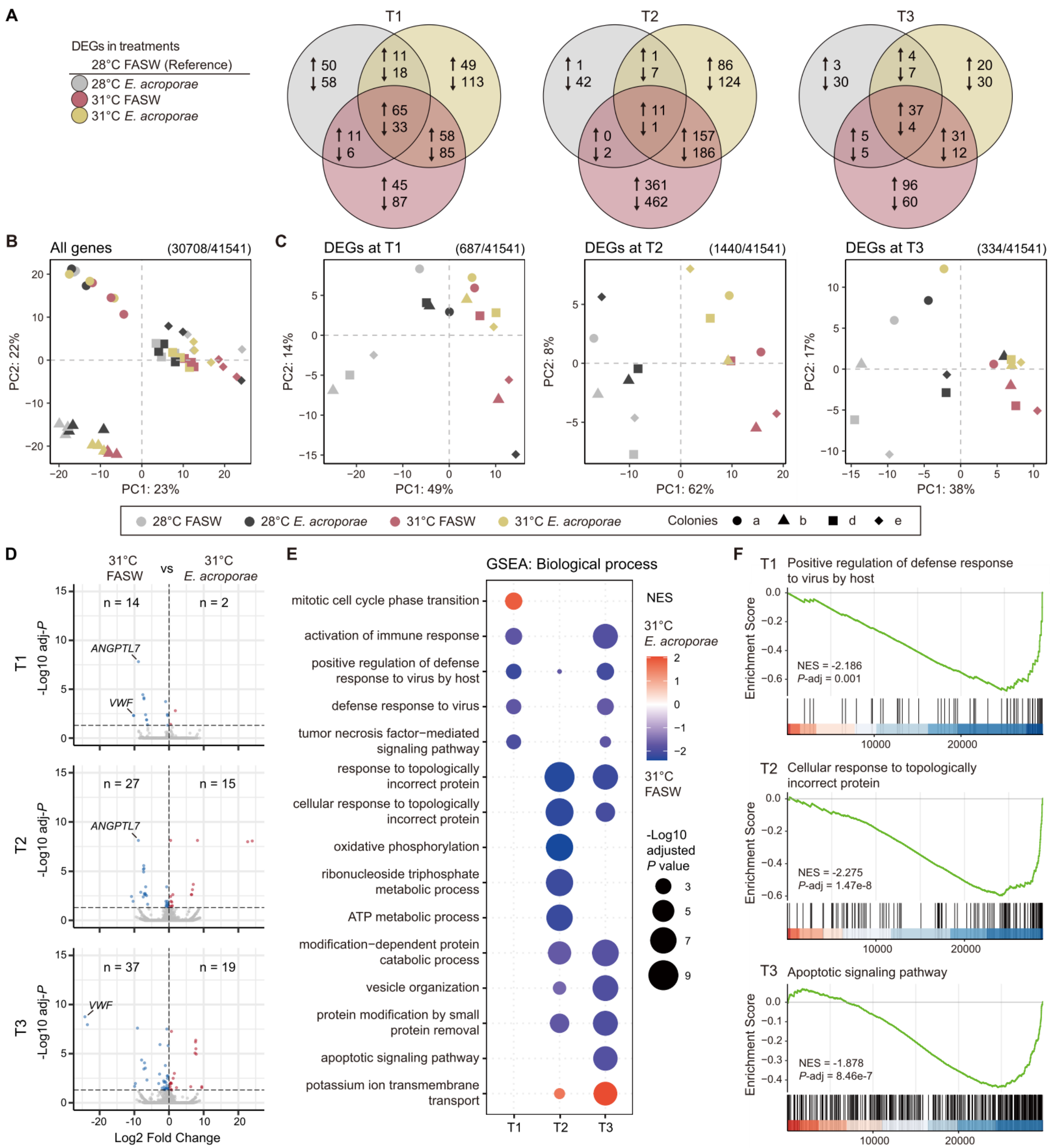

Figure S9. Effects of heat stress and probiotic treatments on the coral host transcriptome. (**A**) Numbers of differentially expressed genes (DEGs) identified in each treatment at each time point, with arrows indicating upregulated and downregulated DEGs (adjusted $P < 0.05$). (**B** and **C**) Principal component analysis (PCA) plots showing coral host transcriptomic profiles, constructed using all pre-filtered genes across all samples (B) or DEGs identified from samples collected at each sampling time point (C). Numbers in parentheses indicate the number of genes used for PCA construction and the total number of genes in the genome-guided (*S. pistillata* clade 1) transcriptome. (**D**) Volcano plots showing results of differential expression analyses comparing the 31°C FASW and 31°C *E. acroporae* treatments. Numbers of upregulated and downregulated

DEGs are indicated by n. (**E**) Gene set enrichment analysis (GSEA) of genes ranked by Wald statistics from DESeq2, comparing the 31°C FASW and 31°C *E. acroporae* treatments. The five most significantly enriched Gene Ontology (GO) terms within the Biological Process category are shown (adjusted $P < 0.05$). (**F**) GSEA running scores for significant GO terms identified at T1, T2, and T3.

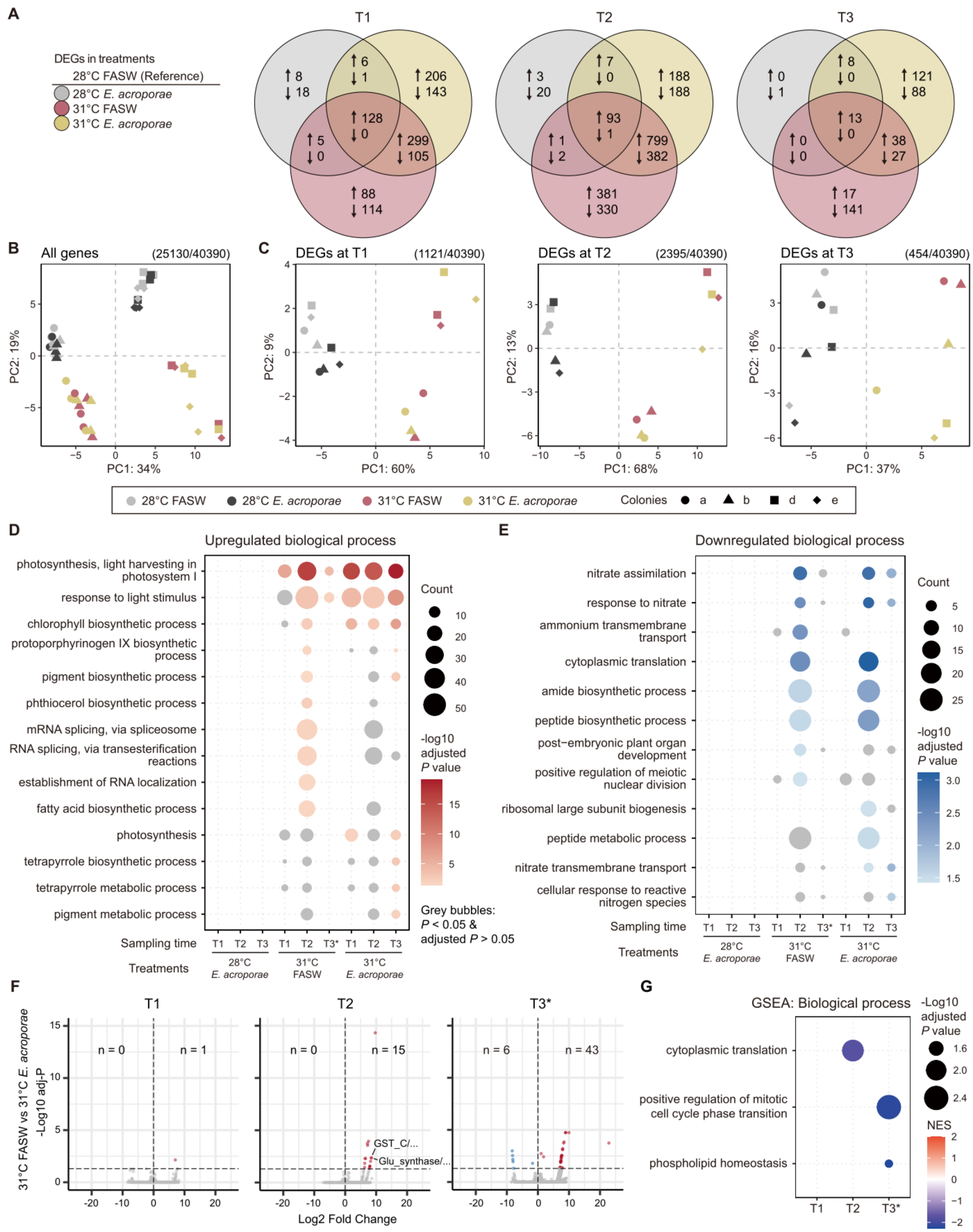

Figure S10. Effects of heat stress and probiotic treatments on the algal symbiont transcriptome. (**A**) Numbers of DEGs identified in algal symbionts from each treatment at each

sampling time point. Arrows indicate upregulated and downregulated DEGs (adjusted $P < 0.05$). (**B** and **C**) PCA plots showing algal symbionts transcriptomic profiles, constructed using all pre-filtered genes across all samples (B) or DEGs identified from samples collected at each sampling time point (C). Numbers in parentheses indicate the number of genes used for PCA construction and the total gene count in the *Cladocopium goreaui* transcriptome. (**D** and **E**) GO enrichment analyses of upregulated (D) and downregulated (E) DEGs identified in algal symbionts across treatments and sampling time points. Bubble size reflects DEG count. Gray bubbles indicate GO term with $P < 0.05$ but adjusted $P > 0.05$ (Benjamini-Hochberg correction). (**F**) Volcano plots showing results of differential expression analyses comparing the 31°C FASW and 31°C *E. acroporae* treatments. Numbers of upregulated and downregulated DEGs are indicated by n. (**G**) GSEA of genes ranked by Wald statistics from DESeq2, comparing the 31°C FASW and 31°C *E. acroporae* treatments. Significantly enriched GO terms (adjusted $P < 0.05$) within the Biological Process category are shown. *Note: Due to severe bleaching, DEGs from the 31°C FASW treatment at T3 were derived from two algal symbiont samples (colonies a and b, n = 2).

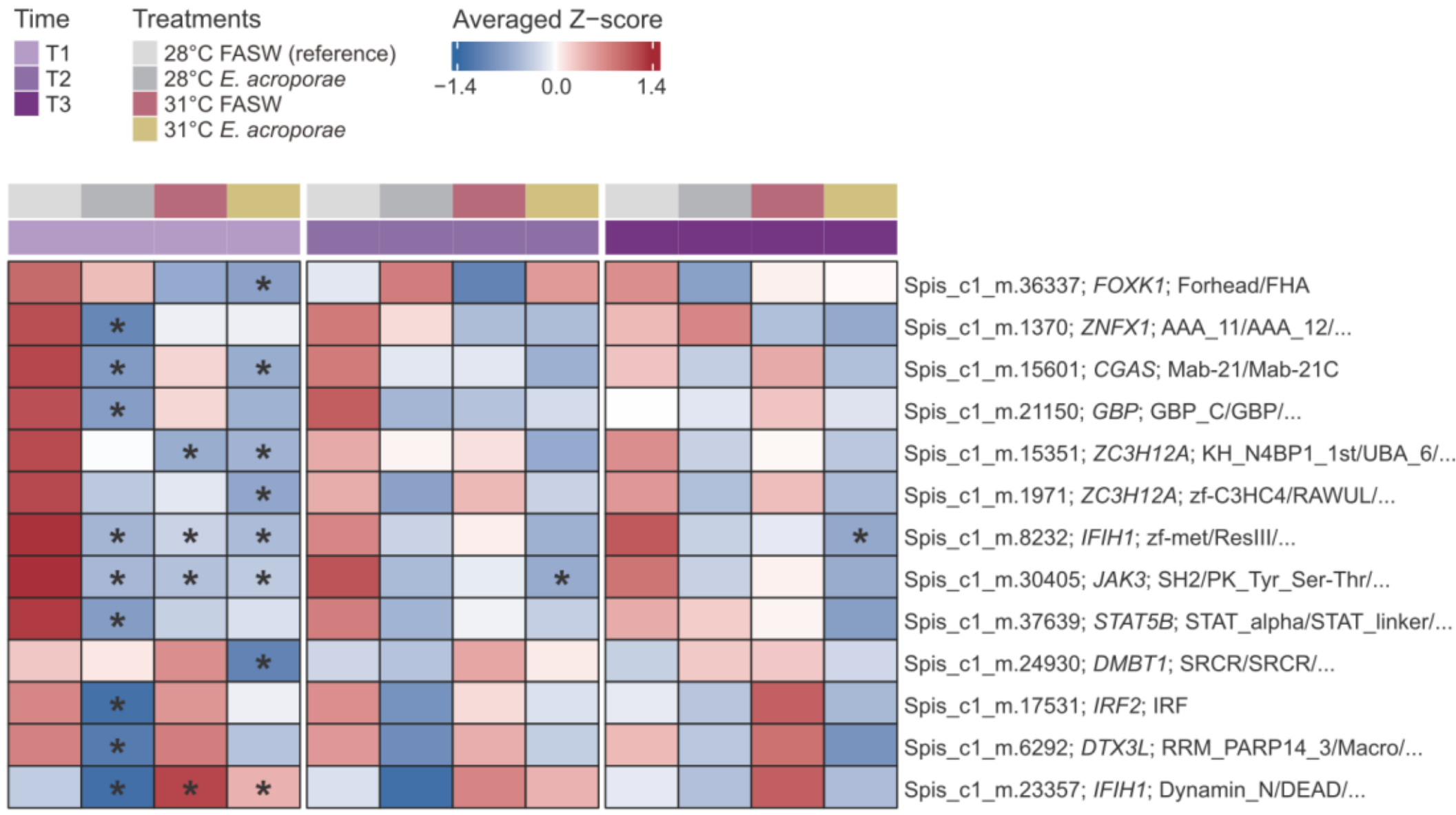

Figure S11. Expression levels of innate immune-associated genes across treatments. Heatmap showing z-scaled bulk transcriptome expression of DEGs identified at T1 within the GO terms “defense response to virus” and “positive regulation of defense response to virus by host.” Asterisks indicate statistical significance (*adjusted $P < 0.05$) based on DESeq2 analysis, with comparisons performed within each time point using the 28°C FASW treatment as the reference group.

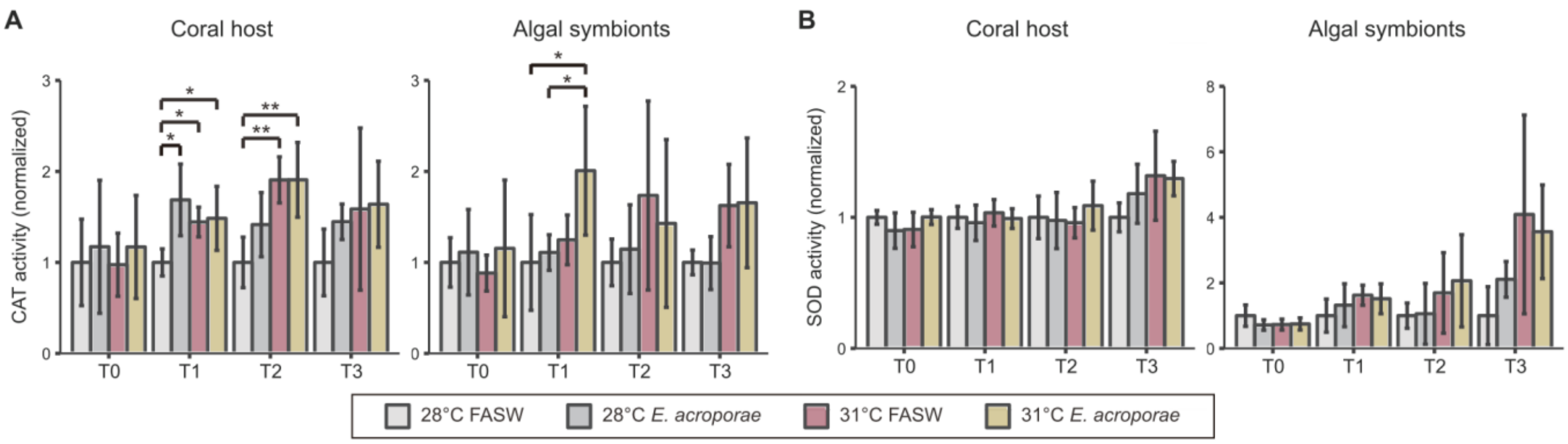

Figure S12. *E. acroporae* enhances catalase activity in the coral host and Symbiodiniaceae. (**A** and **B**) Catalase (A) and superoxide dismutase (B) activities in coral host tissue and algal symbionts across treatments and sampling time points. Enzyme activities were normalized to the control treatment (28°C FASW) at each sampling time point (*adjusted $P < 0.05$, **adjusted $P < 0.01$; n = 5, one-way ANOVA with Tukey's post hoc test).

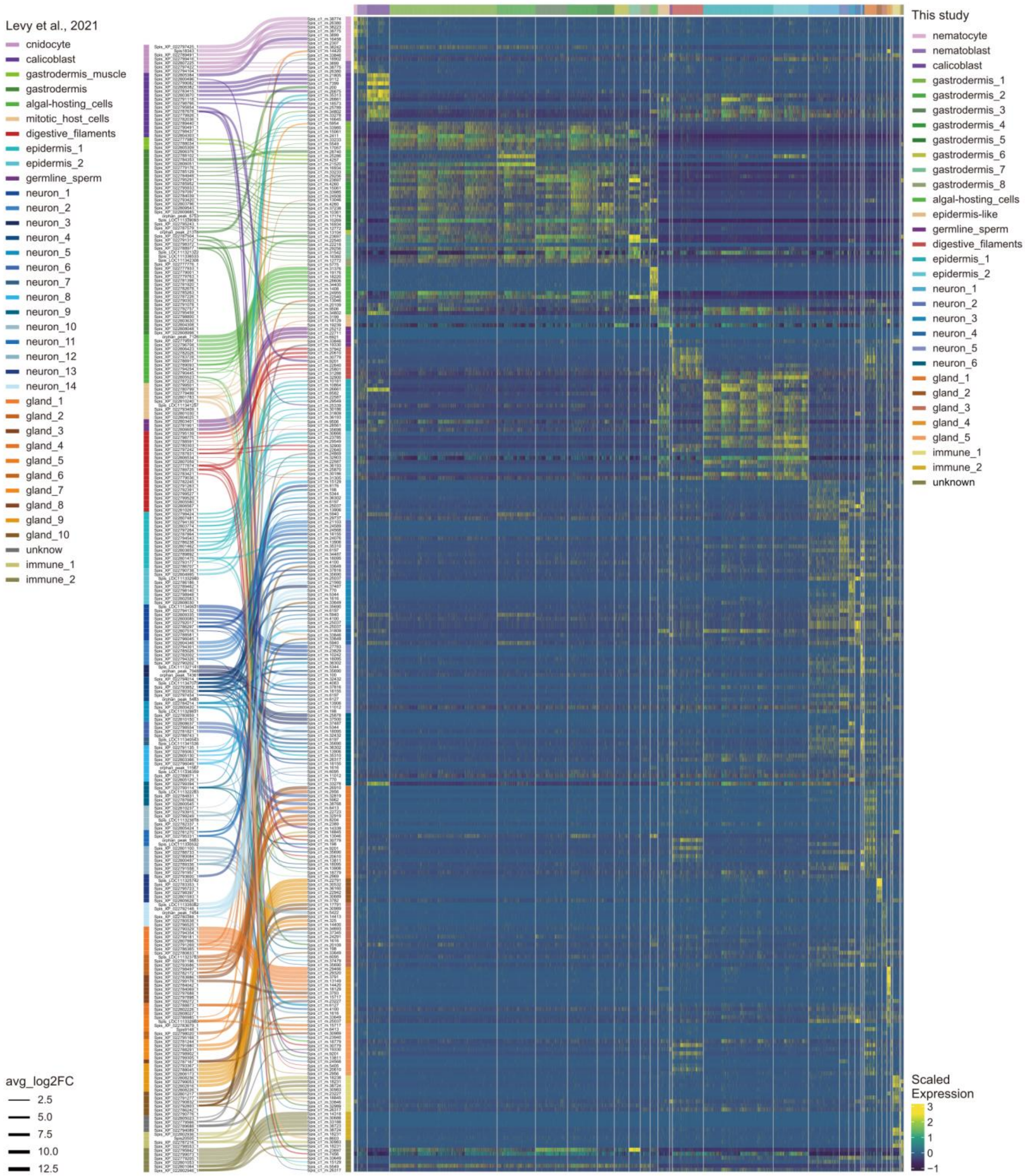

Figure S13. Orthologous markers from *S. pistillata* clade 4 support cell type annotation in clade 1. Bump plot illustrating orthologous marker genes defined between cell-type markers from *S. pistillata* clades 4 and 1. The left column lists 280 marker genes identified in *S. pistillata* clade 4 (see Table S5A). Orthologous markers are connected only to highly variable marker genes identified in *S. pistillata* clade 1 (right column; average log_2 fold change > 1.7; 181 genes in total). Line thickness indicates the average log_2 fold change. Note that marker genes identified in *S.*

pistillata clade 1 are not necessarily exclusive to a single cell cluster and may be shared across multiple clusters). The heatmap shows normalized expression of orthologous marker genes across cells.

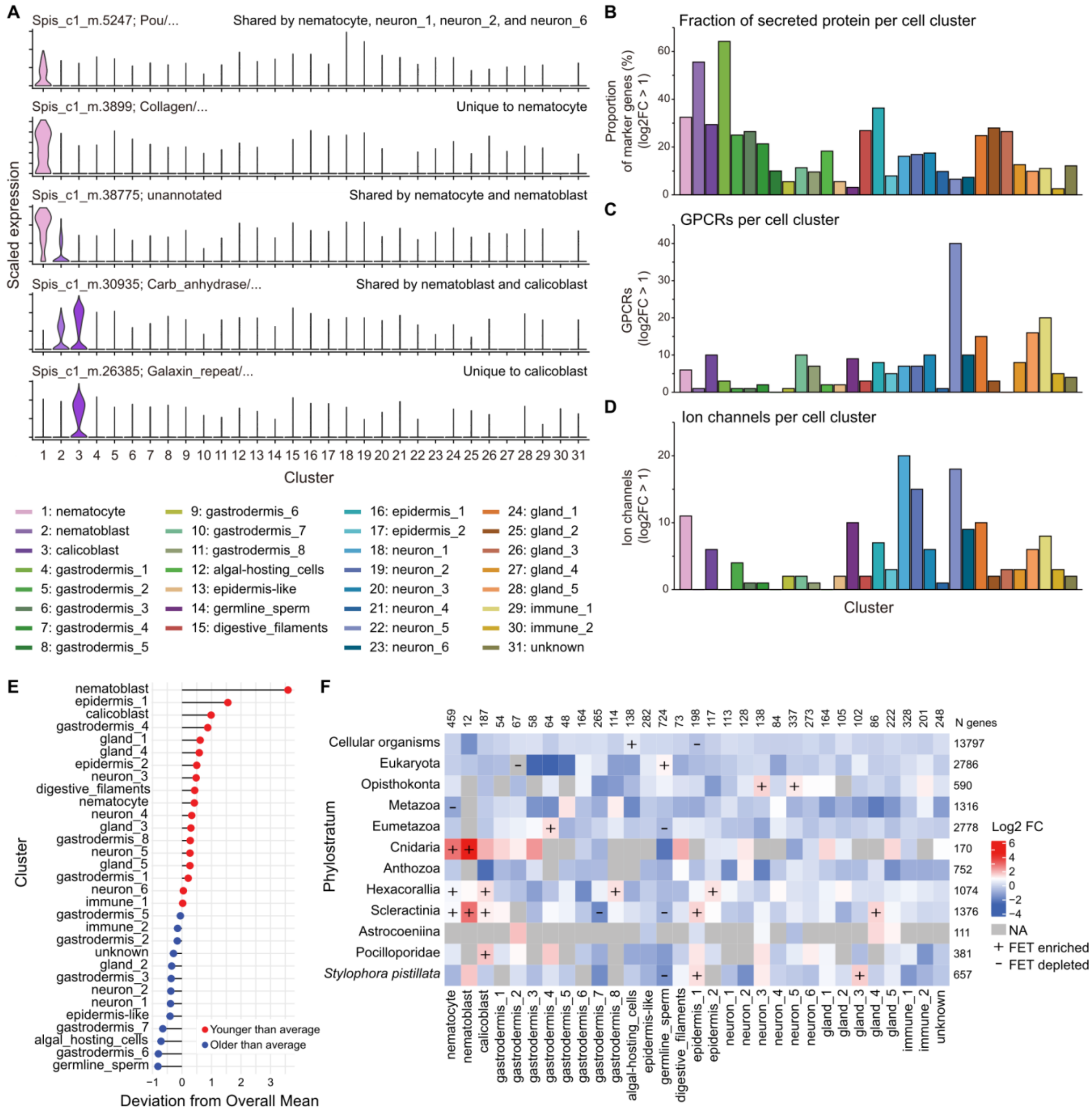

Figure S14. Marker gene functional characteristics and phylostratigraphy of the *S. pistillata* clade 1 cell atlas. (**A**) Expression profiles showing that nematoblasts share multiple marker genes with nematocytes and one marker gene with calicoblasts. (**B**) Percentage of predicted secreted proteins (containing a signal peptide and no transmembrane domains) among marker genes (average $\log_2$ fold change > 1) for each cell cluster. (**C**) Total number of G protein-coupled receptors (GPCRs) genes (containing Pfam domains 7tm_1, 7tm_2, or 7tm_3) identified among marker genes (average $\log_2$ fold change > 1) for each cell cluster. (**D**) Total number of ion channels genes (based on Pfam domains; see Table S6) identified among marker genes (average $\log_2$ fold change > 1) for each cell cluster. (**E**) Mean evolutionary age of marker genes (average $\log_2$ fold change > 1.7) for each cell cluster, as defined by phylostratigraphy. (**F**) Enrichment or depletion of marker genes within each cell cluster across phylostrata. Statistical

significance was determined using Fisher's exact test (adjusted $P < 0.1$, Benjamini-Hochberg correction).

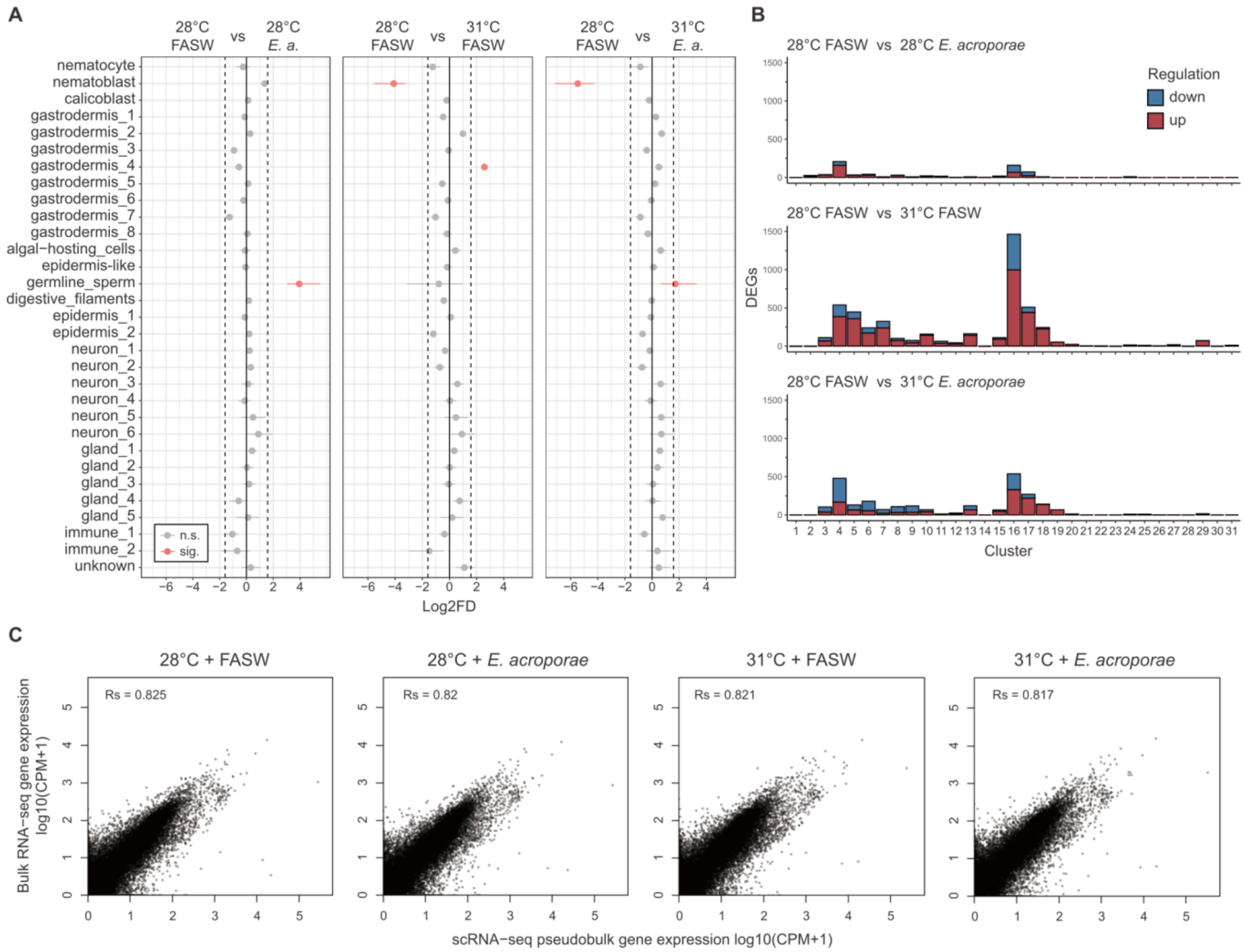

Figure S15. Treatment-induced cellular shifts and concordance between single-cell and bulk transcriptomes. (**A**) Relative differences in cell cluster proportions between the control and each treatment. Red indicates significant changes (FDR < 0.01 and $|Log_2FC| > 1.6$; permutation test). (**B**) Numbers of DEGs (adjusted $P < 0.05$) identified in each cell cluster. (**C**) Gene-wise comparison of pseudobulk scRNA-seq and bulk RNA-seq expression profiles, with Spearman correlation coefficient indicated.

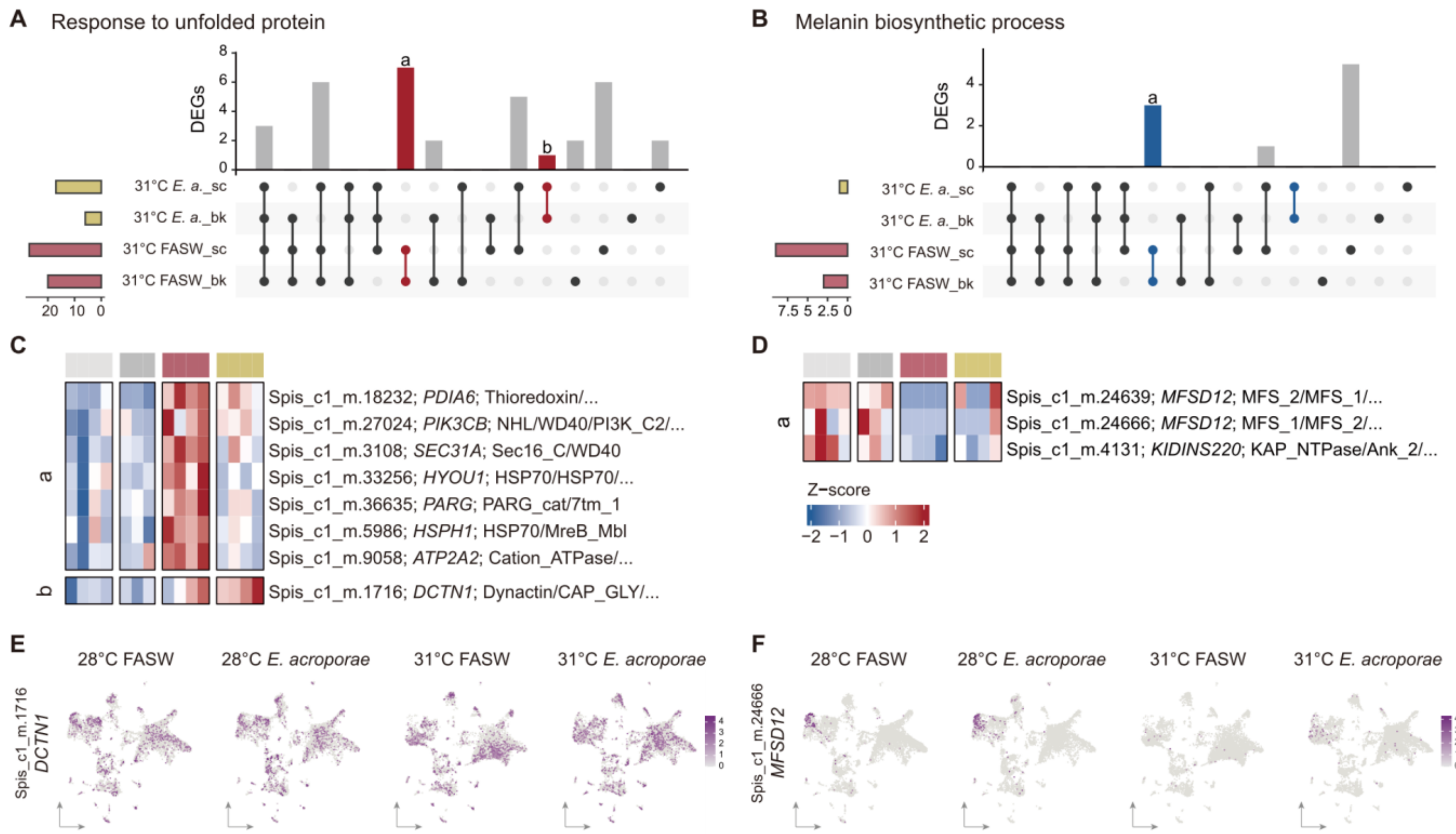

Figure S16. Differential expression of *DCTN1* and *MFSD12* under heat stress with *E. acroporae* treatment. (**A** and **B**) Overlaps of DEGs in the 31°C FASW and 31°C *E. acroporae* treatments (both compared to the 28°C FASW control), associated with GO terms for the response to unfolded protein (A) and the melanin biosynthetic process (B). DEGs identified by bulk RNA-seq ("bk") or single-cell RNA-seq ("sc") are marked. Letters on the bar charts correspond to specific genes detailed in panels (C) and (D), with colors highlighting DEGs uniquely detected in either the 31°C FASW or 31°C *E. acroporae* treatments. (**C** and **D**) Bulk RNA-seq expression of selected genes associated with the response to unfolded protein (C) and the melanin biosynthetic process (D). Annotated gene name and Pfam domains are listed. (**E** and **F**) Single-cell RNA-seq normalized expression of the *DCTN1* (E) and *MFSD12* (F).

Supplementary Table legends

Table S1. Probiotic inoculation optical densities and water parameters. (A) Collected bacterial cultures and inoculation levels measured as optical density at 600 nm. (B) Records of experimental tank water parameters. (C) Statistical details of one-way ANOVA testing the effect of treatment on photosynthetic efficiency (Fv/Fm) at each time point, with Tukey's HSD post hoc tests used for pairwise comparisons.

Table S2. ASV decontamination for 16S rRNA sequencing data. (A) ASVs removed from short-read 16S rRNA gene sequencing data. (B) ASVs removed from long-read 16S rRNA gene sequencing data.

Table S3. Statistical details of the differential abundance analysis of coral microbiota at T0 (A), T1 (B), T2 (C), and T3 (D).

Table S4. Marker genes identified in the *S. pistillata* clade 1 single-cell atlas.

Table S5. *S. pistillata* marker genes identified by reciprocal best hits (RBH) and primer sequences used in this study. (A) *S. pistillata* marker genes identified by RBH. (B) Primer sequences used in this study.

Table S6. Gene annotation of *S. pistillata* clade 1.

Supplementary References

1. Keshavmurthy S, Yang S-Y, Alamaru A *et al.* DNA barcoding reveals the coral "laboratory-rat", stylophora pistillata encompasses multiple identities. *Scientific reports*. 2013;**3**:1520
2. Levy S, Elek A, Grau-Bové X *et al.* A stony coral cell atlas illuminates the molecular and cellular basis of coral symbiosis, calcification, and immunity. *Cell*. 2021;**184**:2973-87. e18
3. Domazet-Lošo T, Brajković J, Tautz D. A phylostratigraphy approach to uncover the genomic history of major adaptations in metazoan lineages. *Trends in Genetics*. 2007;**23**:533-39
4. Grottoli AG, Toonen RJ, van Woesik R *et al.* Increasing comparability among coral bleaching experiments. *Ecological Applications*. 2021;**31**:e02262
5. Liu G, Heron SF, Eakin CM *et al.* Reef-scale thermal stress monitoring of coral ecosystems: New 5-km global products from noaa coral reef watch. *Remote Sensing*. 2014;**6**:11579-606
6. Sheu S-Y, Lin K-R, Hsu M-y *et al.* Endozoicomonas acroporae sp. Nov., isolated from acropora coral. *International journal of systematic and evolutionary microbiology*. 2017;**67**:3791-97
7. Ding J-Y, Shiu J-H, Chen W-M *et al.* Genomic insight into the host–endosymbiont relationship of endozoicomonas montiporae cl-33t with its coral host. *Frontiers in microbiology*. 2016;**7**:251
8. Siebeck U, Marshall N, Klüter A *et al.* Monitoring coral bleaching using a colour reference card. *Coral Reefs*. 2006;**25**:453-60
9. Beers RF, Sizer IW. A spectrophotometric method for measuring the breakdown of hydrogen peroxide by catalase. *J Biol chem*. 1952;**195**:133-40
10. Chen C-P, Tseng C-H, Chen CA *et al.* The dynamics of microbial partnerships in the coral isopora palifera. *The ISME Journal*. 2011;**5**:728-40
11. Jorgensen SL, Hannisdal B, Lanzén A *et al.* Correlating microbial community profiles with geochemical data in highly stratified sediments from the arctic mid-ocean ridge. *Proceedings of the National Academy of Sciences*. 2012;**109**:E2846-E55

12. Yang SH, Lee ST, Huang CR *et al.* Prevalence of potential nitrogen-fixing, green sulfur bacteria in the skeleton of reef-building coral i sopora palifera. *Limnology and oceanography*. 2016;**61**:1078-86
13. Callahan BJ, Wong J, Heiner C *et al.* High-throughput amplicon sequencing of the full-length 16s rrna gene with single-nucleotide resolution. *Nucleic acids research*. 2019;**47**:e103-e03
14. Wada N, Hsu M-T, Tandon K *et al.* High-resolution spatial and genomic characterization of coral-associated microbial aggregates in the coral stylophora pistillata. *Science advances*. 2022;**8**:eabo2431
15. Yamaguchi T, Fuchs BM, Amann R *et al.* Rapid and sensitive identification of marine bacteria by an improved in situ DNA hybridization chain reaction (quickhcr-fish). *Systematic and applied microbiology*. 2015;**38**:400-05
16. Wallner G, Amann R, Beisker W. Optimizing fluorescent in situ hybridization with rrna-targeted oligonucleotide probes for flow cytometric identification of microorganisms. *Cytometry: The Journal of the International Society for Analytical Cytology*. 1993;**14**:136-43
17. Daims H, Brühl A, Amann R *et al.* The domain-specific probe eub338 is insufficient for the detection of all bacteria: Development and evaluation of a more comprehensive probe set. *Systematic and applied microbiology*. 1999;**22**:434-44
18. García-Castro H, Kenny NJ, Iglesias M *et al.* Acme dissociation: A versatile cell fixation-dissociation method for single-cell transcriptomics. *Genome biology*. 2021;**22**:1-34
19. Wang J-T, Chen Y-Y, Tew KS *et al.* Physiological and biochemical performances of menthol-induced aposymbiotic corals. *PLoS ONE*. 2012;**7**:e46406 https://doi.org/https://doi.org/10.1371/journal.pone.0046406
20. Hu Y, Zhang Z, Sun S *et al.* Toward the generation of pure coral genomes with experimental and bioinformatic improvements. *The Innovation*. 2024;**5**
21. Cheng H, Concepcion GT, Feng X *et al.* Haplotype-resolved de novo assembly using phased assembly graphs with hifiasm. *Nature methods*. 2021;**18**:170-75
22. Cheng H, Jarvis ED, Fedrigo O *et al.* Haplotype-resolved assembly of diploid genomes without parental data. *Nature biotechnology*. 2022;**40**:1332-35
23. Cheng H, Asri M, Lucas J *et al.* Scalable telomere-to-telomere assembly for diploid and polyploid genomes with double graph. *Nature methods*. 2024;**21**:967-70
24. Li H. New strategies to improve minimap2 alignment accuracy. *Bioinformatics*. 2021;**37**:4572-74
25. Vasimuddin M, Misra S, Li H *et al.* Efficient architecture-aware acceleration of bwa-mem for multicore systems. *2019 IEEE international parallel and distributed processing symposium (IPDPS)*: IEEE. 314-24.
26. Faust GG, Hall IM. Samblaster: Fast duplicate marking and structural variant read extraction. *Bioinformatics*. 2014;**30**:2503-05
27. Zhou C, McCarthy SA, Durbin R. Yahs: Yet another hi-c scaffolding tool. *Bioinformatics*. 2023;**39**:btac808
28. Durand NC, Shamim MS, Machol I *et al.* Juicer provides a one-click system for analyzing loop-resolution hi-c experiments. *Cell systems*. 2016;**3**:95-98
29. Danecek P, Bonfield JK, Liddle J *et al.* Twelve years of samtools and bcftools. *Gigascience*. 2021;**10**:giab008
30. Durand NC, Robinson JT, Shamim MS *et al.* Juicebox provides a visualization system for hi-c contact maps with unlimited zoom. *Cell systems*. 2016;**3**:99-101
31. Simão FA, Waterhouse RM, Ioannidis P *et al.* Busco: Assessing genome assembly and annotation completeness with single-copy orthologs. *Bioinformatics*. 2015;**31**:3210-12
32. Kuznetsov D, Tegenfeldt F, Manni M *et al.* Orthodb v11: Annotation of orthologs in the widest sampling of organismal diversity. *Nucleic acids research*. 2023;**51**:D445-D51
33. Stanke M, Diekhans M, Baertsch R *et al.* Using native and syntenically mapped cdna alignments to improve de novo gene finding. *Bioinformatics*. 2008;**24**:637-44

34. Stanke M, Schöffmann O, Morgenstern B *et al.* Gene prediction in eukaryotes with a generalized hidden markov model that uses hints from external sources. *BMC bioinformatics*. 2006;**7**:1-11
35. Iwata H, Gotoh O. Benchmarking spliced alignment programs including spaln2, an extended version of spaln that incorporates additional species-specific features. *Nucleic acids research*. 2012;**40**:e161-e61
36. Hoff KJ, Lange S, Lomsadze A *et al.* Braker1: Unsupervised rna-seq-based genome annotation with genemark-et and augustus. *Bioinformatics*. 2016;**32**:767-69
37. Brůna T, Hoff KJ, Lomsadze A *et al.* Braker2: Automatic eukaryotic genome annotation with genemark-ep+ and augustus supported by a protein database. *NAR genomics and bioinformatics*. 2021;**3**:lqaa108
38. Gabriel L, Brůna T, Hoff KJ *et al.* Braker3: Fully automated genome annotation using rna-seq and protein evidence with genemark-etp, augustus, and tsebra. *Genome Research*. 2024;**34**:769-77
39. Buchfink B, Xie C, Huson DH. Fast and sensitive protein alignment using diamond. *Nature methods*. 2015;**12**:59-60
40. Bruna T, Lomsadze A, Borodovsky M. Genemark-etp significantly improves the accuracy of automatic annotation of large eukaryotic genomes. *Genome Research*. 2023;**34**:757-68
41. Pertea G, Pertea M. Gff utilities: Gffread and gffcompare. *F1000Research*. 2020;**9**:ISCB Comm J-304
42. Gotoh O. A space-efficient and accurate method for mapping and aligning cdna sequences onto genomic sequence. *Nucleic acids research*. 2008;**36**:2630-38
43. Kovaka S, Zimin AV, Pertea GM *et al.* Transcriptome assembly from long-read rna-seq alignments with stringtie2. *Genome biology*. 2019;**20**:1-13
44. Gabriel L, Hoff KJ, Brůna T *et al.* Tsebra: Transcript selector for braker. *BMC bioinformatics*. 2021;**22**:1-12
45. Hoff KJ, Lomsadze A, Borodovsky M *et al.* Whole-genome annotation with braker. *Gene prediction: methods and protocols*. 2019:65-95
46. Bolger AM, Lohse M, Usadel B. Trimmomatic: A flexible trimmer for illumina sequence data. *Bioinformatics*. 2014;**30**:2114-20
47. Kim D, Paggi JM, Park C *et al.* Graph-based genome alignment and genotyping with hisat2 and hisat-genotype. *Nature biotechnology*. 2019;**37**:907-15
48. Flynn JM, Hubley R, Goubert C *et al.* Repeatmodeler2 for automated genomic discovery of transposable element families. *Proceedings of the National Academy of Sciences*. 2020;**117**:9451-57
49. Bolyen E, Rideout JR, Dillon MR *et al.* Reproducible, interactive, scalable and extensible microbiome data science using qiime 2. *Nature biotechnology*. 2019;**37**:852-57
50. Martin M. Cutadapt removes adapter sequences from high-throughput sequencing reads. *EMBnet journal*. 2011;**17**:10-12
51. Callahan BJ, McMurdie PJ, Rosen MJ *et al.* Dada2: High-resolution sample inference from illumina amplicon data. *Nature methods*. 2016;**13**:581-83
52. Bokulich NA, Kaehler BD, Rideout JR *et al.* Optimizing taxonomic classification of marker-gene amplicon sequences with qiime 2's q2-feature-classifier plugin. *Microbiome*. 2018;**6**:1-17
53. Quast C, Pruesse E, Yilmaz P *et al.* The silva ribosomal rna gene database project: Improved data processing and web-based tools. *Nucleic acids research*. 2012;**41**:D590-D96
54. Yilmaz P, Parfrey LW, Yarza P *et al.* The silva and "all-species living tree project (ltp)" taxonomic frameworks. *Nucleic acids research*. 2014;**42**:D643-D48
55. Khi Pin C. Nextflow pipeline to analyze pacbio hifi full-length 16s data. https://github.com/PacificBiosciences/pb-16S-nf/tree/b2d55b3464994c1026227d1624758c193d3c2b1e
56. Parks DH, Chuvochina M, Waite DW *et al.* A standardized bacterial taxonomy based on genome phylogeny substantially revises the tree of life. *Nature biotechnology*. 2018;**36**:996-1004

57. Cole JR, Wang Q, Fish JA *et al.* Ribosomal database project: Data and tools for high throughput rrna analysis. *Nucleic acids research*. 2014;**42**:D633-D42
58. McMurdie PJ, Holmes S. Phyloseq: An r package for reproducible interactive analysis and graphics of microbiome census data. *PloS one*. 2013;**8**:e61217
59. Wickham H, Sievert C. *Ggplot2: Elegant graphics for data analysis*, vol. 10: springer New York, 2009.
60. Beule L, Karlovsky P. Improved normalization of species count data in ecology by scaling with ranked subsampling (srs): Application to microbial communities. *PeerJ*. 2020;**8**:e9593
61. Dixon P. Vegan, a package of r functions for community ecology. *Journal of vegetation science*. 2003;**14**:927-30
62. Lin H, Peddada SD. Analysis of compositions of microbiomes with bias correction. *Nature communications*. 2020;**11**:3514
63. Mallick H, Rahnavard A, McIver LJ *et al.* Multivariable association discovery in population-scale meta-omics studies. *PLoS computational biology*. 2021;**17**:e1009442
64. Nearing JT, Douglas GM, Hayes MG *et al.* Microbiome differential abundance methods produce different results across 38 datasets. *Nature communications*. 2022;**13**:342
65. Gu Z, Eils R, Schlesner M. Complex heatmaps reveal patterns and correlations in multidimensional genomic data. *Bioinformatics*. 2016;**32**:2847-49
66. Peschel S, Müller CL, Von Mutius E *et al.* Netcomi: Network construction and comparison for microbiome data in r. *Briefings in bioinformatics*. 2021;**22**:bbaa290
67. Chen CA, Willis BL, Miller DJ. Systematic relationships between tropical corallimorpharians (cnidaria: Anthozoa: Corallimorpharia): Utility of the 5.8 s and internal transcribed spacer (its) regions of the rrna transcription unit. *Bulletin of Marine Science*. 1996;**59**:196-208
68. Tamura K, Stecher G, Kumar S. Mega11: Molecular evolutionary genetics analysis version 11. *Molecular biology and evolution*. 2021;**38**:3022-27
69. Kalyaanamoorthy S, Minh BQ, Wong TK *et al.* Modelfinder: Fast model selection for accurate phylogenetic estimates. *Nature methods*. 2017;**14**:587-89
70. Letunic I, Bork P. Interactive tree of life (itol) v6: Recent updates to the phylogenetic tree display and annotation tool. *Nucleic acids research*. 2024;**52**:W78-W82
71. Marçais G, Delcher AL, Phillippy AM *et al.* Mummer4: A fast and versatile genome alignment system. *PLoS computational biology*. 2018;**14**:e1005944
72. Gosselin S, Fullmer MS, Feng Y *et al.* Improving phylogenies based on average nucleotide identity, incorporating saturation correction and nonparametric bootstrap support. *Systematic biology*. 2022;**71**:396-409
73. Savary R, Barshis DJ, Voolstra CR *et al.* Fast and pervasive transcriptomic resilience and acclimation of extremely heat-tolerant coral holobionts from the northern red sea. *Proceedings of the National Academy of Sciences*. 2021;**118**:e2023298118
74. Sahraeian SME, Mohiyuddin M, Sebra R *et al.* Gaining comprehensive biological insight into the transcriptome by performing a broad-spectrum rna-seq analysis. *Nature communications*. 2017;**8**:59
75. Zardoya R, Costas E, Lopez-Rodas V *et al.* Revised dinoflagellate phylogeny inferred from molecular analysis of large-subunit ribosomal rna gene sequences. *Journal of molecular evolution*. 1995;**41**:637-45
76. Pertea M, Pertea GM, Antonescu CM *et al.* Stringtie enables improved reconstruction of a transcriptome from rna-seq reads. *Nature biotechnology*. 2015;**33**:290-95
77. Mistry J, Chuguransky S, Williams L *et al.* Pfam: The protein families database in 2021. *Nucleic acids research*. 2021;**49**:D412-D19
78. Teufel F, Almagro Armenteros JJ, Johansen AR *et al.* Signalp 6.0 predicts all five types of signal peptides using protein language models. *Nature biotechnology*. 2022;**40**:1023-25
79. Buchfink B, Reuter K, Drost H-G. Sensitive protein alignments at tree-of-life scale using diamond. *Nature methods*. 2021;**18**:366-68

80. Cantalapiedra CP, Hernández-Plaza A, Letunic I *et al.* Eggnog-mapper v2: Functional annotation, orthology assignments, and domain prediction at the metagenomic scale. *Molecular biology and evolution*. 2021;**38**:5825-29
81. Huerta-Cepas J, Szklarczyk D, Heller D *et al.* Eggnog 5.0: A hierarchical, functionally and phylogenetically annotated orthology resource based on 5090 organisms and 2502 viruses. *Nucleic acids research*. 2019;**47**:D309-D14
82. Bryant DM, Johnson K, DiTommaso T *et al.* A tissue-mapped axolotl de novo transcriptome enables identification of limb regeneration factors. *Cell reports*. 2017;**18**:762-76
83. Soneson C, Love MI, Robinson MD. Differential analyses for rna-seq: Transcript-level estimates improve gene-level inferences. *F1000Research*. 2016;**4**:1521
84. Love MI, Huber W, Anders S. Moderated estimation of fold change and dispersion for rna-seq data with deseq2. *Genome biology*. 2014;**15**:1-21
85. Yu G, Wang L-G, Han Y *et al.* Clusterprofiler: An r package for comparing biological themes among gene clusters. *Omics: a journal of integrative biology*. 2012;**16**:284-87
86. Zheng GX, Terry JM, Belgrader P *et al.* Massively parallel digital transcriptional profiling of single cells. *Nature communications*. 2017;**8**:14049
87. Fleming SJ, Chaffin MD, Arduini A *et al.* Unsupervised removal of systematic background noise from droplet-based single-cell experiments using cellbender. *Nature methods*. 2023;**20**:1323-35
88. Hao Y, Stuart T, Kowalski MH *et al.* Dictionary learning for integrative, multimodal and scalable single-cell analysis. *Nature biotechnology*. 2024;**42**:293-304
89. Germain P-L, Lun A, Meixide CG *et al.* Doublet identification in single-cell sequencing data using scdblfinder. *F1000Research*. 2022;**10**:979
90. Miller SA, Policastro RA, Sriramkumar S *et al.* Lsd1 and aberrant DNA methylation mediate persistence of enteroendocrine progenitors that support braf-mutant colorectal cancer. *Cancer research*. 2021;**81**:3791-805
91. Camacho C, Coulouris G, Avagyan V *et al.* Blast+: Architecture and applications. *BMC bioinformatics*. 2009;**10**:1-9
92. Domazet-Lošo T, Tautz D. A phylogenetically based transcriptome age index mirrors ontogenetic divergence patterns. *Nature*. 2010;**468**:815-18
93. Barrera-Redondo J, Lotharukpong JS, Drost H-G *et al.* Uncovering gene-family founder events during major evolutionary transitions in animals, plants and fungi using genera. *Genome Biology*. 2023;**24**:54
94. Conway JR, Lex A, Gehlenborg N. Upsetr: An r package for the visualization of intersecting sets and their properties. *Bioinformatics*. 2017;**33**:2938-40
